# Development of a novel loop-mediated isothermal amplification assay for ß-lactamase gene identification using clinical isolates of Gram-negative bacteria

**DOI:** 10.3389/fcimb.2022.1000445

**Published:** 2023-01-12

**Authors:** Eun Jin Kim, Jiwon Lee, Youngbae Yoon, Donghyun Lee, Yeongjun Baek, Chika Takano, Jun Sakai, Takahiro Iijima, Dai Kanamori, Humphrey Gardner, Robert E. McLaughlin, Paul E. Kilgore, Akihiro Nakamura, Takashi Ogihara, Satoshi Hayakawa, Tomonori Hoshino, Dong Wook Kim, Mitsuko Seki

**Affiliations:** ^1^ Division of Pediatric Dentistry, Department of Human Development and Fostering, Meikai University School of Dentistry, Saitama, Japan; ^2^ Department of Pharmacy, College of Pharmacy, Hanyang University, Ansan, Republic of Korea; ^3^ Institute of Pharmacological Research, Hanyang University, Ansan, Republic of Korea; ^4^ Division of Microbiology, Department of Pathology and Microbiology, Nihon University School of Medicine, Tokyo, Japan; ^5^ Department of Infectious Disease and Infection Control, Saitama Medical University, Saitama, Japan; ^6^ Harbour Biomed, Natick, MA, United States; ^7^ Institute for Life Science Entrepreneurship, Union, NJ, United States; ^8^ Department of Pharmacy Practice, Eugene Applebaum College of Pharmacy & Health Sciences, Wayne State University, Detroit, MI, United States

**Keywords:** loop-mediated isothermal amplification, ß-lactamase gene, Gram-negative bacteria, *bla*_KPC_, *bla*_NDM-1_, *bla*_IMP-1_ group, *bla*_VIM_

## Abstract

Rapid evaluation of antimicrobial susceptibility is important in the treatment of nosocomial infections by Gram-negative bacteria, which increasingly carry carbapenemases and metallo-β-lactamases. We developed loop-mediated isothermal amplification (LAMP)-based assays for four β-lactamase genes (*bla*_KPC_, *bla*_NDM-1_, *bla*_IMP-1_ group, and *bla*_VIM_). The assays were evaluated using eight reference bacterial strains (*Klebsiella pneumoniae*, *Escherichia coli*, *Pseudomonas aeruginosa*, and *Acinetobacter bereziniae*) harboring six β-lactamase genes. A total of 55 Gram-negative bacterial strains, including 47 clinical *P. aeruginosa* isolates, fully characterized by next-generation sequencing (NGS), were used to evaluate the LAMP assays. The results were compared to those of conventional PCR. The LAMP assays were able to detect as few as 10 to 100 copies of a gene, compared to 10 to 10^4^ copies for conventional PCR. The LAMP assay detected four β-lactamase genes with a sensitivity similar to that using purified DNA as the template in DNA-spiked urine, sputum, and blood specimens. By contrast, the sensitivity of PCR was 1- to 100-fold lower with DNA-spiked clinical specimens. Therefore, the LAMP assays were proved to be an appropriate tool for the detection of four β-lactamases.

## Introduction

Antimicrobial resistance (AMR) is a serious public health problem globally (Laxminarayan et al., 2016). Increased use of antimicrobial drugs promotes the emergence of antimicrobial-resistant bacterial strains and related infections. Some of those bacteria have become resistant to several antibiotics, including carbapenems and third-generation cephalosporins (Arumugham et al., 2022; Palacios-Baena et al., 2021)—the best-available antibiotics for treating infections by multidrug-resistant bacteria. In 2017, the World Health Organization (WHO) published a global priority list of antibiotic-resistant bacteria to guide research, discovery, and the development of new antibiotics (WHO, 2017). These critical multidrug-resistant bacteria include carbapenem-resistant *Acinetobacter baumannii*, carbapenem-resistant *Pseudomonas aeruginosa*, and carbapenem-resistant/third-generation cephalosporin-resistant *Enterobacteriaceae* (including *Klebsiella pneumoniae, Escherichia coli*, and *Enterobacter* spp.). These have marked effects on mortality and healthcare.

Carbapenemases are categorized into Ambler class A, B, and D β-lactamases. Ambler classes A and D are serine proteases while class B enzymes are metallo-β-lactamases (MBL). *Klebsiella pneumoniae* carbapenemase (*bla*_KPC_) is plasmid-encoded and is the most prevalent class A carbapenemase worldwide, including in the United States (Chen et al., 2014). New Delhi metallo-β-lactamase (*bla*_NDM_), imipenem-resistant *Pseudomonas* type carbapenemase (*bla*_IMP_), and Verona integron-encoded metallo-β-lactamase (*bla*_VIM_) are class B carbapenemases that can be transmitted among different strains of the same bacterial species and among different bacterial species or genera (WHO, 2020b). The transmission of resistance can occur by transformation (bacteria take up DNA from the environment), transduction (DNA transfer between bacterial cells by bacteriophages), conjugation (plasmids harboring mobile elements), and the transfer of plasmids between bacterial cell *via* outer membrane vesicles. Thus, in a hospital, several bacterial species may have the same resistance determinants which were transferred by mobile genetic elements.

AMR, especially in Gram-negative bacteria, is complex, has a variety of underlying mechanisms and links to diseases in humans and other animals (WHO, 2017). The resistance of common bacteria, including *P. aeruginosa* and *Enterobacteriaceae*, is rendering treatments for common infections ineffective, worsening outcomes and increasing healthcare costs (WHO, 2020a). Global laboratory-based surveillance for AMR is now critical to understand the spread of multidrug resistant organisms as well as evaluate the impact of interventions designed to reduce the clinical and community burden of these dangerous pathogens.

Rapid, simple, and reliable identification of AMR is essential to ensure that antibiotic use is appropriate, and for surveillance in low- or middle-income countries. Carbapenem resistance is examined by calculating the minimum inhibitory concentration (MIC) using the disc diffusion test, gradient method, or synergy test (double-disc test), but these do not clarify the mechanism of drug resistances. Molecular assays are the standard tests for the identification of carbapenemase-encoding genes (Solanki et al., 2014).

Genotype-based methods (*i.e.*, PCR, real-time PCR, and microarrays) can subgroup carbapenemases and monitor the transmission of resistance. Conventional PCR-based assays can detect β-lactamase genes but require well-equipped laboratories. However, the equipment required for conventional PCR or real-time PCR assays is expensive, and these techniques are complex, hampering their adoption by laboratories with limited experience in molecular testing, particularly in low- or middle-income countries (WHO, 2019).

The loop-mediated isothermal amplification (LAMP) assay overcomes the limitations of phenotyping and PCR methods (Mori and Notomi, 2019). This method uses a unique priming mechanism that yields specific DNA products more rapidly than PCR. It does not require expensive equipment or a sophisticated laboratory. LAMP is convenient in terms of point-of-care testing (POCT). For surveillance, it is appropriate for laboratories lacking experience with molecular testing. We developed LAMP assays for four β-lactamase genes (*bla*_KPC_, *bla*_NDM-1_, *bla*_IMP-1_ group, and *bla*_VIM_) and evaluated each using clinical strains isolated at diverse geographical locations (Kos et al., 2015). There are several previous reports mentioning the LAMP assay for the four β-lactamase genes identification (Moreno-Morales et al., 2020; Feng et al., 2021). While these reports used isolates from a limited number of regions, this is the first report to use clinical strains isolated at diverse geographical locations. In addition, phylogenetical analysis verified the extent of its efficacy. This is in stark contrast to previous reports that simply referred to effectiveness.

## Materials and methods

### Bacterial strains

A total of 63 bacterial strains, 8 reference and 55 clinical were used to develop the LAMP assays. The reference strains harboring six β-lactamase genes (*bla*_KPC_, *bla*_NDM-1_, *bla*_IMP_, *bla*_VIM_, *bla*_OXA-48_, and *bla*_GES_) were three *K. pneumoniae*, one *E. coli*, three *P. aeruginosa*, and one *Acinetobacter bereziniae* strains; all were provided by AstraZeneca (Waltham, MA) (**Table 1**).

**Table 1:** Reactivities and specificities of LAMP assays.

Strain ID	Species	Genotype	LAMP Assays ^a^			
			*bla*_KPC_	*bla*_NDM-1_	*bla*_IMP-1_	*bla*_VIM_
ARC2945	*K. pneumoniae*	KPC-2	(+) ^b^	(-)	(-)	(-)
ARC2929	*K. pneumoniae*	KPC-3	(+)	(-)	(-)	(-)
ARC3600	*E. coli*	NDM-1	(-)	(+)	(-)	(-)
ARC3802	*K. pneumoniae*	NDM-1	(-)	(+)	(-)	(-)
ARC2780	*A. bereziniae*	IMP-1	(-)	(-)	(+)	(-)
ARC3471	*P. aeruginosa*	VIM-2	(-)	(-)	(-)	(+)
ARC3475	*P. aeruginosa*	OXA-48	(-)	(-)	(-)	(-)
ARC3917	*P. aeruginosa*	GES-1	(-)	(-)	(-)	(-)

a LAMP results determined via Loopamp real-time turbidimetry and the naked eye.

b (-), negative; (+), positive.

### Preparation of chromosomal DNA

Genomic DNA was extracted using a Maxwell 16-Cell DNA Purification kit (Promega, Madison, WI). The concentration of purified genomic DNA was determined using a NanoDrop 1000 Spectrophotometer (Thermo Fisher Scientific Inc., Waltham, MA). The genomic DNA copy number was calculated based on genome size (5.4 Mbp for *K. pneumoniae* [Kp52.154, GenBank accession number: FO834906.1], 5.2 Mbp for *E. coli* [CFT073, AE014075.1], 6.5 Mbp for *P. aeruginosa* [PB369, CP025049.1], 4.5 Mbp for *A. bereziniae* [XH901, NZ_CP018259.1], and 4.0 Mbp for *A. baumannii* [ATCC19606, CP059040.1]).

The eight standard reference strains including seven β-lactamase genes (**Table 1**) were used to evaluate the specificity of the LAMP assay for each targeted β-lactamase gene. The specificity of each LAMP reactions was examined using 10^5^ copies of reference genomic DNA per reaction. A serial 10- times dilution of reference genomic DNA was used to test the sensitivity of the LAMP assays in comparison to that of PCR.

### Clinical Gram-negative bacterial strains

Fifty-five clinical Gram-negative isolates harboring β-lactamase genes were used to evaluate the LAMP assays (**Table 2**). Among them, 47 clinical *P. aeruginosa* strains were randomly selected from 388 strains of known genotypes and phenotypes (Kos et al., 2015) isolated at diverse geographical locations (Colombia, India, Spain, France, Greece, Germany, Argentina, Croatia, China, Brazil, Mexico, Romania, and the Philippines) between 2003 and 2012. Originally, those isolates were obtained from the International Health Management Association (IHMA). Whole-genome sequences were analyzed on the HiSeq 2000 or MiSeq platform (Illumina, San Diego, CA, United States) (Kos et al., 2015). Susceptibility to meropenem was explored using frozen Trek-Sensititre custom plates (Thermo Fisher Scientific Inc.) following the 2012 guidelines of the Clinical and Laboratory Standards Institute (Clinical and Laboratory Standards Institute, 2012). The MICs for meropenem are listed in **Table 2**. Other eight clinical Gram-negative isolates including three *A. baumannii*, two *E. coli*, two *K. pneumoniae*, and one *E. cloacae* were obtained from AstraZeneca culture collection (**Table 2**).

**Table 2:** Clinical isolates evaluated.

Strain ID	Species	Origin of isolate		Genotype	Meropenem		LAMP Assays			
		Country	Anatomical site		MIC (mg/L)	Susceptibility	*bla*_KPC_	*bla*_NDM-1_	*bla*_IMP-1 group_	*bla*_VIM_
KPC, NDM-1, IMP-1 group, and VIM ß-lactamase-producing strains										
AZPAE14719	*P. aeruginosa*	Colombia	RTI^a^	KPC-2	>32	(R)^d^	(+)	(-)	(-)	(-)
AZPAE14720	*P. aeruginosa*	Colombia	UTI	KPC-2, OXA-2	>32	(R)	(+)	(-)	(-)	(-)
AZECO 3801	*E. coli*	–	–	NDM-1, OXA-1, TEM-1, CTX-M-15	>8	(R)	(-)	(+)	(-)	(-)
AZKPN 4770	*K. pneumoniae*	–	–	NDM-1, OXA-1, TEM-1, CTX-M-15	>8	(R)	(-)	(+)	(-)	(-)
AZABA 5986	*A. baumannii*	–	–	NDM-1,OXA-10, OXA-23, OXA-69	>8	(R)	(-)	(+)	(-)	(-)
AZECL 5127	*E. cloacae*	–	–	IMP-1, OXA-10, OXA-48, TEM-1	>8	(R)	(-)	(-)	(+)	(-)
AZPAE14702	*P. aeruginosa*	Philippines	RTI	IMP-4, OXA-10	>32	(R)	(-)	(-)	(+)	(-)
AZPAE14703	*P. aeruginosa*	Philippines	IAI	VIM-1, OXA-10	>32	(R)	(-)	(-)	(-)	(+)
AZPAE14811	*P. aeruginosa*	India	RTI	VIM-2, OXA-4	>32	(R)	(-)	(-)	(-)	(+)
AZPAE14922	*P. aeruginosa*	France	RTI	VIM-2, OXA-1	>32	(R)	(-)	(-)	(-)	(+)
AZPAE14929	*P. aeruginosa*	Germany	UTI	VIM-2, OXA-4	>32	(R)	(-)	(-)	(-)	(+)
AZPAE14958	*P. aeruginosa*	India	IAI	VIM-2, OXA-10	>32	(R)	(-)	(-)	(-)	(+)
AZPAE14984	*P. aeruginosa*	France	UTI	VIM-2, OXA-4	>32	(R)	(-)	(-)	(-)	(+)
AZPAE15029	*P. aeruginosa*	France	RTI	VIM-2, OXA-4	>32	(R)	(-)	(-)	(-)	(+)
AZPAE14706	*P. aeruginosa*	Greece	IAI	PSE-1, VIM-4, OXA-35	>32	(R)	(-)	(-)	(-)	(+)
AZPAE14865	*P. aeruginosa*	India	RTI	VEB-like, VIM-5, OXA-10	>32	(R)	(-)	(-)	(-)	(+)
AZPAE14900	*P. aeruginosa*	India	IAI	VEB-like, VIM-5, OXA-10	16	(R)	(-)	(-)	(-)	(+)
AZPAE13879	*P. aeruginosa*	Argentina	–	VIM-11, OXA-17	16	(R)	(-)	(-)	(-)	(+)
Other 37 ß-lactamase-producing strains including IMP-13, 15 and 18, and VIM-7										
AZPAE14862	*P. aeruginosa*	India	UTI	IMP-13	2	(S)	(-)	(-)	(-)	(-)
AZPAE13872	*P. aeruginosa*	Mexico	–	IMP-15	>32	(R)	(-)	(-)	(-)	(-)
AZPAE14688	*P. aeruginosa*	Mexico	–	IMP-18	>32	(R)	(-)	(-)	(-)	(-)
AZPAE 3936	*P. aeruginosa*	–	–	VIM-7	–	–	(-)	(-)	(-)	(-)
AZPAE13848	*P. aeruginosa*	India	–	GES-9	0.25	(S)	(-)	(-)	(-)	(-)
AZPAE13856	*P. aeruginosa*	India	–	GES-7	0.5	(S)	(-)	(-)	(-)	(-)
AZPAE13880	*P. aeruginosa*	Mexico	–	OXA-2, GES-19	>32	(R)	(-)	(-)	(-)	(-)
AZPAE14694	*P. aeruginosa*	Romania	UTI	PER-1, OXA-2, OXA-74	16	(R)	(-)	(-)	(-)	(-)
AZPAE14708	*P. aeruginosa*	Greece	IAI	OXA-19	16	(R)	(-)	(-)	(-)	(-)
AZPAE14819	*P. aeruginosa*	Brazil	UTI	SPM-1, OXA-56	>32	(R)	(-)	(-)	(-)	(-)
AZPAE14821	*P. aeruginosa*	Brazil	UTI	SPM-1, OXA-56	>32	(R)	(-)	(-)	(-)	(-)
AZPAE14822	*P. aeruginosa*	Brazil	IAI	OXA-56	8	(R)	(-)	(-)	(-)	(-)
AZPAE14831	*P. aeruginosa*	Argentina	RTI	GES-1	0.5	(S)	(-)	(-)	(-)	(-)
AZPAE14834	*P. aeruginosa*	Argentina	UTI	OXA-2	8	(R)	(-)	(-)	(-)	(-)
AZPAE14838	*P. aeruginosa*	China	RTI	OXA-10	8	(R)	(-)	(-)	(-)	(-)
AZPAE14846	*P. aeruginosa*	France	RTI	PSE-1	32	(R)	(-)	(-)	(-)	(-)
AZPAE14852	*P. aeruginosa*	Brazil	RTI	OXA-17	32	(R)	(-)	(-)	(-)	(-)
AZPAE14853	*P. aeruginosa*	Brazil	RTI	SPM-1, OXA-56	>32	(R)	(-)	(-)	(-)	(-)
AZPAE14886	*P. aeruginosa*	Croatia	UTI	PSE-1	16	(R)	(-)	(-)	(-)	(-)
AZPAE14887	*P. aeruginosa*	Croatia	IAI	OXA-2	32	(R)	(-)	(-)	(-)	(-)
AZPAE14912	*P. aeruginosa*	Croatia	RTI	OXA-2	32	(R)	(-)	(-)	(-)	(-)
AZPAE14923	*P. aeruginosa*	Brazil	RTI	OXA-56, SPM-1	>32	(R)	(-)	(-)	(-)	(-)
AZPAE14926	*P. aeruginosa*	Brazil	UTI	AER-like	16	(R)	(-)	(-)	(-)	(-)
AZPAE14933	*P. aeruginosa*	France	UTI	OXA-9, PSE-1	0.25	(S)	(-)	(-)	(-)	(-)
AZPAE14948	*P. aeruginosa*	Argentina	IAI	GES-5	>32	(R)	(-)	(-)	(-)	(-)
AZPAE14956	*P. aeruginosa*	Germany	IAI	VEB-1, OXA-10	16	(R)	(-)	(-)	(-)	(-)
AZPAE14976	*P. aeruginosa*	China	RTI	PSE-1	32	(R)	(-)	(-)	(-)	(-)
AZPAE14983	*P. aeruginosa*	Croatia	RTI	PSE-1	16	(R)	(-)	(-)	(-)	(-)
AZPAE15000	*P. aeruginosa*	Spain	UTI	OXA-2	16	(R)	(-)	(-)	(-)	(-)
AZPAE15002	*P. aeruginosa*	Spain	IAI	OXA-46, OXA-101	>32	(R)	(-)	(-)	(-)	(-)
AZPAE15047	*P. aeruginosa*	Argentina	RTI	TEM-1	>32	(R)	(-)	(-)	(-)	(-)
AZPAE15054	*P. aeruginosa*	Colombia	UTI	OXA-2	8	(R)	(-)	(-)	(-)	(-)
AZPAE15063	*P. aeruginosa*	Brazil	RTI	CTX-M-2, OXA-129	1	(S)	(-)	(-)	(-)	(-)
AZABA 2675	*A. baumannii*	–	–	OXA-113	–	–	(-)	(-)	(-)	(-)
AZABA 2782	*A. baumannii*	–	–	OXA-23, OXA-66, TEM-1, PER-1	–	–	(-)	(-)	(-)	(-)
AZECO 4089	*E. coli*	–	–	OXA-48	<=1	(S)	(-)	(-)	(-)	(-)
AZKPN 4593	*K. pneumoniae*	–	–	OXA-48	>8	(R)	(-)	(-)	(-)	(-)

^a^RTI, Respiratory tract infections; UTI, Urinary tract infections; IAI, Intra-abdominal infections.

^b^(S), susceptible; (R), resistant.

### LAMP primer design

To design primers for *bla*_KPC_, *bla*_NDM_, *bla*_IMP_, and *bla*_VIM_, we compared the sequences of variants using Clustal X v. 2.0 (Larkin et al., 2007) to identify consensus regions and to select the target region. The GenBank accession numbers and names of the *bla*_KPC_, *bla*_NDM_, *bla*_IMP_, and *bla*_VIM_ sequences are listed in **Supplementary Table 1**. The alignments of the four target genes are shown in **Supplementary Figure 1**.

LAMP primers were designed using Primer Explorer v. 5 software according to the sequences of *bla*_KPC_ (GenBank accession number NG_049253.1), *bla*_NDM-1_ (FN396876.1), *bla*_IMP-1_ group (GU831546.1), and *bla*_VIM_ (GQ853417.1) (**Supplementary Figure 2**). Nucleotide 22 of the backward inner primer (BIP) for *bla*_VIM_ was changed from G to C to increase the sensitivity and prevent primer dimer formation (**Table 3**).

**Table 3:** LAMP primer sequences in this study.

Primer name	LAMP primer Sequence (Sequence 5’-3’)	Length (base pairs)	Gene/Genbank no.	Reaction temperature
KPC_F3	TGT ACG CGA TGG ATA CCG G	19	*bla*_KPC/_ NG_049253	65°C
KPC_B3	CAC CGT CAT GCC TGT TGT	18		
KPC_FIP	CAG CAC AGC GGC AGC AAG AAA TGT AAG TTA CCG CGC TGA GG	41		
KPC_BIP	GGC TTG CTG GAC ACA CCC ATT TTC CGA GAT GGG TGA CCA C	40		
KPC_LF	GCC CTT GAA TGA GCT	15		
KPC_LB	CGT TAC GGC AAA AAT GCG C	19		
NDM-1_F3	TGC ATG CCC GGT GAA ATC C	19	*bla*_NDM-1_/ FN396876	63°C
NDM-1_B3	TCA TCG GTC CAG GCG GTA T	19		
NDM-1_FIP	GAG CTG GCG GAA AAC CAG ATC GAC GAT TGG CCA GCA AAT	39		
NDM-1_BIP	ATG TCT GGC AGC ACA CTT CCG CCA TCC CTG ACG ATC AAA C	40		
NDM-1_LF	AAC CGT TGG TCG CCA GTT T	19		
NDM-1_LB	TTT CGG GGC AGT CGC TT	17		
IMP-1_F3	CCG GGA CAC ACT CCA GAT	18	*bla*_IMP-1_/ GU831546	65°C
IMP-1_B3	GTT TCA AGA GTG ATG CGT CTC C	22		
IMP-1_FIP	CAC CCA AAT TGC CTA AAC CGT CGT AGT GGT TTG GTT GCC TG	41		
IMP-1_BIP	AGA AGC TTG GCC AAA GTC CGT GGA ACA ACC AGT TTT GCC TTA	42		
IMP-1_LF	CCA CCG AAT AAT ATT TTC CT	20		
IMP-1_LB	CCA AAT TAT TAA AGT CCA AAT ATG G	25		
VIM_F3	CGT GAT GGT GAT GAG TTG CT	20	*bla*_VIM-2_/ GQ853417	67°C
VIM_B3	TCG TTC CCC TCT ACC TCG	18		
VIM_FIP	CGC GTT ACA GGA AGT CCA AGG GTG CGA AAA ACA CAG C	37		
VIM_BIP	CTC CAC GCA CTT TCA TGA CGG **C** ^a^TG ATG CGT ACG TTG CC	38		
VIM_LF	TTT GCT TCT CAA TCT CCG	18		
VIM_LB	TTG ATG TCC TTC GGG C	16		

a original sequence was G (to avoid formation of primer dimer and nonspecific reactions).

Based on the primer sequences, we aligned primers’ binding sites of variants of each β-lactamase gene, also phylogenetic trees were conducted by the neighbor-joining method using MEGA v. 11 (Tamura et al., 2021) (**Supplementary Figure 3**). According to an *in silico* analysis (FastPCR (PrimerDigital, 2022)), variants in the blue square in **Supplementary Figure 3** were expected to be detected by the LAMP assays.

### LAMP reaction

The LAMP reaction mixture (25 μL) contained 1.6 μM FIP and BIP, 0.2 μM F3 and B3, 0.8 μM LF and LB, 8 U of *Bst* Polymerase (large fragment) (New England Biolabs, Ipswich, MA), 1.4 mM four deoxynucleoside triphosphates, 0.8 M betaine (Sigma, St. Louis, MO), 20 mM Tris-HCl (pH 8.8), 10 mM KCl, 10 mM (NH_4_)_2_SO_4_, 8 mM MgSO_4_, 0.1% (v/v) Tween 20, and template DNA (2 μL) (Takano et al., 2019). Reactions were incubated at 63-67°C for 60 min (**Table 3**) and heated at 80°C for 2 min to terminate the reaction.

### Analysis of LAMP products

The LAMP reaction was monitored by measuring turbidity in real time using Loopamp^®^ Real-Time Turbidimeters (LA-500 and LA-200; Eiken Chemical Co., Tokyo, Japan) at a wavelength of 650 nm (OD_650_) at 6 s intervals. The reaction time was recorded when the turbidity level exceeded 0.1 in accordance with the manufacturer’s protocol. Amplified products were also evaluated visually. Amplified products were sequenced at the Akita Prefectural University Biotechnology Center using a BigDye^®^ Terminator v. 3.1 Cycle Sequencing Kit and 3130xL Genetic Analyzer (Applied Biosystems, Foster City, CA). The F2 and B2 primers used to sequence the target regions are shown in **Supplementary Table 2**.

### PCR

The PCR primers and conditions for the β-lactamases (**Supplementary Table 3**) were described previously (Senda et al., 1996; Monteiro et al., 2012). PCR assays were performed in a 25 μL reaction mixture containing 0.2 mM each deoxynucleoside triphosphate, 10 mM Tris-HCl (pH 8.0), 10 mM KCl, 2.5 mM MgCl_2_, 0.4 μM each primer, 2.5 U of Ex Taq DNA Polymerase (Takara, Shiga, Japan), and template DNA (2 μL). Reactions were performed on a SimpliAmp™ Thermal Cycler (Applied Biosystems). The PCR program was 5 min at 94°C; followed by 35 cycles of denaturation at 94°C for 90 s, annealing at 55°C for 30 s, and extension at 72°C; and a final extension at 72°C for 15 min, followed by storage at 4°C. The PCR products were subjected to agarose gel electrophoresis and visualized with Midori Green Advance (NIPPON Genetics, Tokyo, Japan).

### DNA-spiked specimens

To analyze the effects of biological substances on the established LAMP assays, the detection limits of the LAMP assays were determined using DNA-spiked clinical samples. Urine and blood specimens were collected from five healthy volunteers at Nihon University School of Medicine. The blood specimens were heparinized and stored at -80°C. Sputum specimens were obtained from seven patients at Ageo Central General Hospital and frozen at -80°C to inactivate bacteria. After approval by the Biosafety Committee of Nihon University, the specimens were handled using the risk group 2 protocol of the Laboratory Biosafety Manual of the WHO, Geneva, 2004. Urine specimens were boiled at 95°C for 5 min and centrifuged at 1500 rpm. DNA was extracted from blood and sputum samples using a Loopamp™ PURE DNA Extraction Kit (Eiken Chemical Co.) according to the manufacturer’s instructions. Purified β-lactamase DNA (*bla*_KPC_, ARC2945; *bla*_NDM-1_, ARC3600; *bla*_IMP-1_ group, ARC2780; and *bla*_VIM_, ARC3471) was added to the specimens and used to determine the detection limits of the PCR and LAMP assays.

## Results

### Analytical reactivity and specificity of the β-lactamase LAMP assay

**Figure 1:**
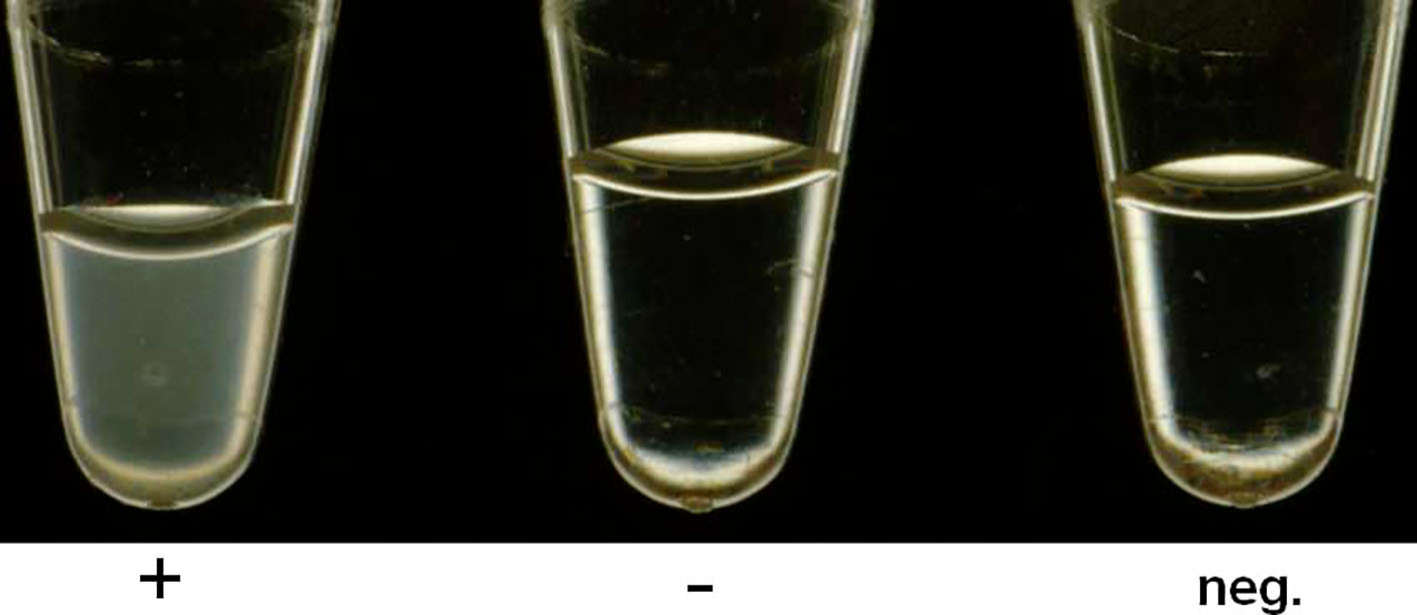
*bla*_KPC_-LAMP assay detecting Visual detection of a white precipitate in positive samples. The figure shows a positive (*bla*_KPC-2_) and a negative (non-*bla*_KPC_, *bla*_NDM-1_) sample, together with a negative control (neg.).

The LAMP assays amplified the target sequences, as confirmed by turbidity in the reaction tube and by real-time turbidimetry (**Table 1** and **Figure 1**). Of the eight strains including 7 β-lactamase genes, each assay detected only the target gene. In contrast, no other genes were not amplified in this assay (**Table 1**). The LAMP reactions reached the detection threshold (turbidity level of 0.1) within 35 min, whereas no turbidity rise was detected for 60 min in negative reaction. The sequences of the LAMP products were identical to those expected (**Supplementary Figures 2, 4**).

### Analytical sensitivity of the β-lactamase LAMP assay

Serial 10-fold-diluted DNA samples (adjusted at 10^4^, 10^3^, 10^2^, 10, and 1, and 0 genome copies per reaction in 25 μL) were amplified by LAMP and the results were compared to those of PCR. The detection limits of the LAMP assays were 10^2^ copies per reaction for *bla*_KPC_, 10^2^ copies for *bla*_NDM-1_, 10^2^ copies for *bla*_IMP-1_, and 10 copies for *bla*_VIM_. The detection limits for PCR were 10^4^ copies for *bla*_KPC_, 10^3^ copies for *bla*_NDM-1_, 10^2^ copies for *bla*_IMP_, and ten copies for *bla*_VIM_ (**Table 4**). Therefore, the LAMP assays were up to 100-fold more analytically sensitive than PCR. Although the detection limits of the LAMP assays for *bla*_IMP-1_ and *bla*_VIM_ were identical to those of PCR, the LAMP reaction was more rapid, and the results could be confirmed visually.

**Table 4:** Detection limits of the PCR and LAMP assays used to detect *bla*_KPC_, *bla*_NDM-1_, *bla*_IMP-1_ and *bla*_VIM_ in DNA-spiked specimens.

	Detection limit							
	*bla*_KPC_		*bla*_NDM-1_		*bla*_IMP-1_		*bla*_VIM_	
	PCR	LAMP	PCR	LAMP	PCR	LAMP	PCR	LAMP
Purified DNA	10^4^ copies^a^	10^2^	10^3^	10^2^	10^2^	10^2^	10	10
DNA spiked specimens								
Urine^b^	10^4^	10^2^	10^4^	10^2^	10^2^	10^2^	10^2^	10
Sputum^c^	10^4^	10^2^	10^4^	10^2^	10^2^	10^2^	10^2^	10
Blood^c^	10^4^	10^2^	10^4^	10^2^	10^4^	10^2^	10^3^	10

a Amount of DNA per reaction;

b Supernatant data obtained after boiling and centrifugation;

c Samples prepared via Loopamp™ PURE DNA extraction kit (Eiken Chemical Co.).

### DNA-spiked specimens

The detection limit of the LAMP assays using clinical specimens was determined using DNA-spiked urine, sputum, and blood samples. The detection limit of the LAMP assays was 10^2^ copies per reaction for *bla*_KPC_, 10^2^ copies for *bla*_NDM-1_, 10^2^ copies for *bla*_IMP-1_, and 10 copies for *bla*_VIM_ in DNA-spiked urine, sputum, and blood specimens. These values were identical to those obtained using purified DNA as the template (**Table 4**).

The detection limit of PCR for *bla*_KPC_ in DNA-spiked urine, sputum, and blood specimens was 10^4^ copies per reaction, identical to that using purified DNA as the template. The detection limit of PCR for *bla*_NDM-1_ was 10^4^ copies per reaction in DNA-spiked urine, sputum, and blood specimens, which was 10-fold lower than when purified DNA was used as the template. The detection limit of PCR for *bla*_IMP_ in DNA-spiked urine and sputum specimens was 10^2^ copies, identical to that using purified DNA as a template. However, the sensitivity decreased 100-fold (10^4^ copies per reaction) for the DNA-spiked blood specimens compared to using purified DNA as the template. Finally, the detection limit of PCR for *bla*_VIM_ in DNA-spiked urine and sputum specimens was 10-fold less sensitive (10^2^ copies per reaction) and that in DNA-spiked blood specimens was 100-fold less sensitive (10^3^ copies per reaction) compared to the use of purified DNA as the template (10 copies per reaction). The sensitivity of LAMP for DNA-spiked blood specimens was up to 100-fold higher than that of PCR.

### Evaluation of LAMP assays for clinical Gram-negative bacterial strains

The LAMP assays for four β-lactamases were validated using 55 clinical Gram-negative bacterial strains isolated at diverse geographical locations (**Table 2**). The LAMP assay for *bla*_KPC_ specifically amplified the target *bla*_KPC_ segments (*bla*_KPC-2_) and no other genotype. The LAMP assay for *bla*_NDM-1_ specifically amplified three isolates with the target gene *bla*_NDM-1_, and no other genotype. The LAMP assay for *bla*_IMP-1_ amplified two isolates with *bla*_IMP-1_ and *bla*_IMP-4_, but not three isolates with *bla*_IMP-13_, *bla*_IMP-15_, and *bla*_IMP-18_ (**Table 2**) or any other genotype. The LAMP assay for *bla*_VIM_ amplified 11 isolates with *bla*_VIM-1_, *bla*_VIM-2_, *bla*_VIM-4_, *bla*_VIM-5_, and *bla*_VIM-11_, but not an isolate with *bla*_VIM-7_ (**Table 2**) or any other genotype. The results of the LAMP assays for *bla*_KPC_, *bla*_NDM-1_, *bla*_IMP-1_, and *bla*_VIM_ were thus identical to those simulated *in silico* (blue squares in **Supplementary Figure 3**).

## Disscussion

We developed LAMP assays for four β-lactamase genes (*bla*_KPC_, *bla*_NDM-1_, *bla*_IMP-1_ group, and *bla*_VIM_). Standard reference strains and 55 β-lactamase-carrying clinical isolates were correctly identified, although most Metallo β-lactamase genes have sequence variations and 47 clinical *P. aeruginosa* isolates were from diverse geographical locations. Theoretically, the capacity of detection will not change if the sequence of the genes is the same; however, the genome size of *P. aeruginosa*, co-existing drug-resistant genes, and the phenotype of the isolates are different depending on geographic distribution (Kos et al., 2015). Thus, the investigation using diverse geographic locations is important to assess the quality of the assay.

The results were in agreement with those of our in silico simulations. The sensitivity of the LAMP assays was comparable between purified DNA and DNA-spiked clinical specimens. The LAMP reactions were not inhibited by contaminants in DNA-spiked samples. By contrast, PCR assays for three β-lactamase genes (*bla*_NDM-1_, *bla*_IMP-1_ group, and *bla*_VIM_) are inhibited by the contaminants. The LAMP assays were up to 100-fold more sensitive than PCR. Because of their robustness to biological substances, the LAMP assays detected DNA added to blood specimens with up to 100-fold higher sensitivity than PCR.

PCR assays are affected by, for example, heparin (Satsangi et al., 1994), heme, leukocyte DNA, and IgG (Al-Soud et al., 2000; Al-Soud and Radstrom, 2001). Therefore, it is not appropriate to use a simple DNA extraction method such as the Loopamp™ PURE DNA Extraction Kit (Eiken Chemical Co.), which is a contamination-resistant kit that does not require a centrifuge or refrigerator. Because of their robustness to biological substances, the LAMP assays can be performed at the bedside or resource-limited settings. The LAMP assays will promote the control of β-lactam resistance because rapid and accurate POCT for drug resistance is essential and will facilitate research on AMR and the effectiveness of the *One Health* approach to limit the spread of resistance (WHO, 2017).

In this study, we struggled to detect sequence variations for each of the MBL genotypes. Most MBL genes have sequence variations. Indeed, 93 variants of *bla*_KPC_, 40 of *bla*_NDM_, 87 of *bla*_IMP_, and 77 variants of *bla*_VIM_ have been reported (**Supplementary Table 1**). To select the target region, we compared the sequences of the four genotypes, and identified a consensus region of each genotype (**Supplementary Figure 1**). The 93 *bla*_KPC_ variants do not differ markedly and all were expected to be detected by the LAMP assay (**Supplementary Figures 1, 3**). The 40 *bla*_NDM_ variants also do not differ markedly and all were expected to be detected by the LAMP assay (**Supplementary Figures 1, 3**). *bla*_IMP_ has 87 highly different variants, which prevented identification of a consensus region. Therefore, we focused on the *bla*_IMP-1_ group, which is main group among the variants of *bla*_IMP_ (**Supplementary Figures 1, 3**). Among the 77 *bla*_VIM_ variants, we identified a consensus region for 73; however, 4 variants (*bla*_VIM-7_, *bla*_VIM-18_, *bla*_VIM-61_, and *bla*_VIM-69_) were considerably different (**Supplementary Figures 1, 3**). Therefore, we focused on the 73 *bla*_VIM_ variants as targets for the LAMP assays.

We designed LAMP primers for four target genes (nucleotides 141 to 359 of *bla*_KPC_, 76 to 287 of *bla*_NDM-1_, 463 to 676 of *bla*_IMP-1_, and 223 to 446 of *bla*_VIM_) (**Supplementary Figure 2**). The results agreed with those of *in silico* simulations (blue squares in **Supplementary Figure 3**). Using the LAMP primers for the *bla*_IMP-1_ group, *bla*_IMP-1_ and *bla*_IMP-4_ (blue square in **Supplementary Figure 3**) were detected but *bla*_IMP-13_, *bla*_IMP-15_, *bla*_IMP-18_ (without the blue square in **Supplementary Figure 3**) were not. The LAMP primers for *bla*_VIM_ detected *bla*_VIM-1, 2, 4, 5, and 11_ (blue square in **Supplementary Figure 3**), but not *bla*_VIM-7_ (without the blue square in **Supplementary Figure 3**). The LAMP primers for *bla*_KPC_ detected *bla*_KPC-2_ and *bla*_KPC-3_ (blue square in **Supplementary Figure 3**), and that for *bla*_NDM-1_ detected *bla*_NDM-1_ (blue square in **Supplementary Figure 3**).

Visual inspection of LAMP assay is sufficient to confirm positive results. For more complex diagnostic assays, such as arrays and whole-genome sequencing, complex analyses are needed to interpret the raw data. Indeed, LAMP-based diagnostic tests for extended-spectrum β-lactamases and carbapenemases are suitable for widespread use, including in low- or middle-income countries.

This study had a several limitations. The specificity of LAMP assays for *bla*_IMP_ and *bla*_VIM_ does not cover all of the genotypes. If MBL is suspected clinically but negative results are obtained, additional qualification should be considered, e.g., sequencing. Additional work using clinical specimens from patients is required.

In conclusion, we developed novel LAMP assays for four β-lactamase genes (*bla*_KPC_, *bla*_NDM-1_, *bla*_IMP-1_ group, and *bla*_VIM_). Further evaluation of these assays is required using additional clinical specimens. Low-complexity and low-cost tests may be suitable for most types of laboratories, whereas high-complexity and high-cost tests may be suitable only for established national reference laboratories with a sufficient budget. Laboratories with no prior experience in molecular testing may consider low-complexity, low-cost tests such as LAMP-based assays. Further development of additional LAMP primer sets for *bla*_VIM_ and *bla*_IMP_ is now ongoing.

## Data availability statement

The datasets presented in this study can be found in online repositories. The names of the repository/repositories and accession number(s) can be found in the article/**Supplementary Material**.

## Ethics statement

The studies involving human participants were reviewed and approved by the Ethics Committees of Ageo Central General Hospital (approval # 434) and the IRB of Nihon University School of Medicine (approval # 28-9-0). Using the IRB-approved protocol, seven patient sputum specimens (Ageo Central General Hospital) were collected in accordance with the recommendations of the Japan Society of Clinical Examination Medicine. This guidance enables access to specimens when it is difficult to obtain consent, the sample is anonymized, and if the IRB has approved the study protocol. The requirement for written consent was waived because specimens were anonymized samples discarded by the Hospital’s clinical laboratory. We also used urine and blood specimens from five healthy volunteers at Nihon University School of Medicine. The study protocol was reviewed and approved by the Institutional Review Board (IRB) of the Nihon University School of Medicine (# 28-9-0). Written informed consent was obtained from the five healthy volunteers. The patients/participants provided their written informed consent to participate in this study.

## Author contributions

HG, RM, PK, SH, TH, DK, and MS contributed the conception of this study. EK, DK and MS designed the experiments. EK, JL, YY, DL, YB, TI, and MS performed the experiments. HG, RM, CT, SH and PK acquired the samples. EK, JL, JS, CT and MS analyzed the data. EK, JS, TI, DK, AN, TO, TH, and MS performed the phylogenetic and in silico analyses. EK, JS, MS, DK, and SH interpreted the data, drafted the manuscript. All authors contributed to the article and approved the submitted version.
