# Supplementary material for: Development of a novel loop-mediated isothermal amplification assay for ß-lactamase gene identification using clinical isolates of Gram-negative bacteria
Source: Front Cell Infect Microbiol. 2023 Jan 12;12:1000445. doi: 10.3389/fcimb.2022.1000445 (PMC9877619; doi:10.3389/fcimb.2022.1000445)
Supplement: Supplementary file 1 [file DataSheet_1.pdf]

Table S1. Names and GenBank accession numbers of reference strains carrying the four β-lactamase genes (*bla*$_{KPC}$, *bla*$_{NDM}$, *bla*$_{IMP}$, and *bla*$_{VIM}$). Information on the reference strains was obtained from the National Center for Biotechnology Information (https://www.ncbi.nlm.nih.gov/). The following variants do not exist: *bla*$_{KPC}$ (1, 83, 89, 92, 93); *bla*$_{NDM}$ (32, 33); *bla*$_{IMP}$ (36, 50, 57); *bla*$_{VIM}$ (21, 22).

blaKPC	Strain Name	Organism Name	GenBank No.
2	-	*Klebsiella pneumoniae*	NG_049253.1
3	CL-5761	*Klebsiella pneumoniae*	NG_049257.1
4	KpAM1	*Klebsiella pneumoniae*	NG_049258.1
5	PR280	*Pseudomonas aeruginosa*	NG_049259.1
6	Kp-421	*Klebsiella pneumoniae*	NG_049260.1
7	-	*Klebsiella pneumoniae*	NG_049261.1
8	S2KP8-1	*Klebsiella pneumoniae*	NG_049262.1
9	P3000136	*Escherichia coli*	FJ624872.1
10	C1AC9-24	*Acinetobacter baumannii*	NG_049243.1
11	Kp10-26	*Klebsiella pneumoniae*	NG_049244.1
12	ZJ10620	*Klebsiella pneumoniae*	NG_049245.1
13	EN038	*Enterobacter cloacae*	NG_049246.1
14	BK13048	*Klebsiella pneumoniae*	NG_049247.1
15	Kp1241	*Klebsiella pneumoniae*	NG_049248.1
16	MDRKP-288	*Klebsiella pneumoniae*	NG_049249.1
17	MDRKP-209	*Klebsiella pneumoniae*	NG_049250.1
18	746649	*Escherichia coli*	NG_049251.1
19	AM MDR 53	*Klebsiella pneumoniae subsp. pneumoniae*	NG_049252.1
20	15-50575E	*Escherichia coli*	MF772496
21	INSRA19829	*Escherichia coli*	NG_049254.1
22	KP1211	*Klebsiella pneumoniae*	NG_049255.1
23	1728750	*Klebsiella pneumoniae*	NG_060569.1
24	UC331	*Klebsiella pneumoniae*	NG_049256.1
25	JMI#21	*Klebsiella pneumoniae*	NG_051167.1
26	BT_746	*Klebsiella pneumoniae*	NG_051469.1
27	KPC-079	*Klebsiella pneumoniae*	NG_052862.1
28	DAC	*Escherichia coli*	NG_052581.1
29	1427927	*Klebsiella pneumoniae*	NG_055580.1
30	1472816	*Klebsiella pneumoniae*	NG_054685.1
31	1526(3-C)	*Klebsiella pneumoniae*	NG_055494.1
32	1431(1-C)	*Klebsiella pneumoniae*	NG_055495.1
33	NR5632	*Klebsiella pneumoniae*	NG_056170.1
34	db2927	*Klebsiella pneumoniae*	NG_057447.1
35	15-32-006	*Klebsiella pneumoniae*	NG_060524.1
36	1751543	*Klebsiella pneumoniae*	NG_061389.1
37	CRE-1026	*Klebsiella pneumoniae*	NG_061612.1
38	ST17	*Klebsiella pneumoniae*	NG_062357.1
39	FER	*Klebsiella pneumoniae*	NG_063841.1
40	1-RC-17-044091-1	*Enterobacter hormaechei*	NG_064726.1
41	UM	*Klebsiella pneumoniae*	NG_065876.1
42	NRZ-46298	*Klebsiella pneumoniae*	NG_064727.1
43	SECR19-0437	*Klebsiella pneumoniae*	NG_064728.1
44	3/2018	*Klebsiella pneumoniae*	NG_065427.1
45	1856530	*Enterobacter cloacae*	NG_065877.1
46	1818312	*Klebsiella pneumoniae*	NG_065878.1
47	183860	*Klebsiella pneumoniae*	NG_074714.1
48	183358	*Klebsiella pneumoniae*	NG_074715.1
49	Ec2R	*Escherichia coli*	NG_071203.1
50	N859	*Klebsiella pneumoniae*	NG_068507.1
51	1B	*Klebsiella pneumoniae*	NG_067224.1
52	7B	*Klebsiella pneumoniae*	NG_067225.1
53	LC-1825/18	*Klebsiella pneumoniae subsp. Pneumoniae*	NG_068176.1
54	SECR19-2960	*Klebsiella pneumoniae*	NG_067226.1
55	BS407	*Klebsiella pneumoniae*	NG_068177.1
56	SENTRY Antimicrobial Surveillance Program 941690	*Klebsiella pneumoniae*	NG_068016.1
57	KP759	*Klebsiella pneumoniae subsp. Pneumoniae*	NG_068508.1
58	1106489	*Klebsiella pneumoniae*	NG_070177.1
59	1099927	*Klebsiella pneumoniae*	NG_070178.1
60	NRZ-56857	*Escherichia coli*	NG_070179.1
61	K53	*Klebsiella pneumoniae*	NG_070180.1
62	KP-12R	*Klebsiella pneumoniae*	NG_073465.1
63	KP-19R	*Klebsiella pneumoniae*	NG_073466.1
64	KP-32R	*Klebsiella pneumoniae*	NG_073467.1
65	KP-38R	*Klebsiella pneumoniae*	NG_073468.1
66	1959651	*Klebsiella pneumoniae*	NG_070739.1
67	04068429-17	*Klebsiella pneumoniae*	NG_074716.1
68	01258927-20	*Klebsiella pneumoniae*	NG_074717.1
69	03108465-40	*Klebsiella pneumoniae*	NG_074718.1
70	02288527-42	*Klebsiella pneumoniae*	NG_074719.1
71	KP697	*Klebsiella pneumoniae*	NG_070895.1
72	2000213	*Klebsiella pneumoniae*	NG_070740.1
73	2006689	*Klebsiella pneumoniae*	NG_070741.1
74	KP55	*Klebsiella pneumoniae*	NG_070742.1
75	130053	*Klebsiella pneumoniae*	NG_070743.1
76	HS453	*Klebsiella pneumoniae*	NG_070896.1
77	090305X2B1	*Klebsiella pneumoniae*	NG_070897.1
78	KP HX1016	*Klebsiella pneumoniae*	NG_071204.1
79	HS2953	*Klebsiella pneumoniae*	NG_071205.1
80	M25399	*Klebsiella pneumoniae*	NG_073469.1
81	M25752	*Klebsiella pneumoniae*	NG_073470.1
82	MRSN755319	*Citrobacter koseri*	NG_073471.1
84	130120	*Klebsiella pneumoniae*	NG_074720.1
85	RIVM_C016661	*Klebsiella pneumoniae*	NG_074721.1
86	Y2	*Klebsiella pneumoniae*	NG_074722.1
87	S10	*Klebsiella pneumoniae*	NG_074723.1
88	S207	*Klebsiella pneumoniae*	NG_074724.1
90	2216735	*Klebsiella pneumoniae*	NG_076666.1
91	2220020	*Klebsiella pneumoniae*	NG_076667.1
94	-	*Klebsiella pneumoniae*	NG_076680.1
95	-	*Klebsiella pneumoniae*	NG_076681.1
96	M25197	*Klebsiella pneumoniae*	OK086970.1
97	M25923	*Klebsiella pneumoniae*	OK086971.1
98	FMUL503	*Klebsiella pneumoniae*	MZ893466.1

blaNDM	Strain Name	Organism Name	GenBank No.
1	pKpANDM-1	*Klebsiella pneumoniae*	NG_049326.1
2	MK	*Acinetobacter baumannii*	NG_049334.1
3	QECAU3	*Escherichia coli*	NG_049335.1
4	I5	*Escherichia coli*	NG_049336.1
5	EC405	*Escherichia coli*	NG_049337.1
6	1100101	*Escherichia coli*	NG_049338.1
7	N11-02566	*Escherichia coli*	NG_049339.1
8	NCGM37	*Escherichia coli*	NG_049340.1
9	PPH1303	*Klebsiella pneumoniae*	NG_049341.1
10	kpn/78	*Klebsiella pneumoniae*	NG_049327.1
11	KnPEc14	*Escherichia coli*	NG_049328.1
12	IOMTU 388.1	*Escherichia coli*	NG_049329.1
13	IOMTU 558	*Escherichia coli*	NG_049330.1
14	JN49-1	*Acinetobacter lwoffii*	NG_049331.1
15	EN5134	*Escherichia coli*	NG_049332.1
16a	1121740	*Klebsiella pneumoniae*	NG_049333.1
16b	TA8571	*Escherichia coli*	NG_074726.1
17	AD-19R	*Escherichia coli*	NG_052662.1
18	NRZ-30964	*Escherichia coli*	NG_052866.1
19	N17-00233	*Escherichia coli*	NG_055498.1
20	CCD1	*Escherichia coli*	NG_057455.1
21	SCEC020023	*Escherichia coli*	NG_055664.1
22	1700882	*Escherichia coli*	NG_057612.1
23	1631112	*Klebsiella pneumoniae*	NG_060570.1
24	1670925	*Providencia stuartii*	NG_060571.1
25	18AR0740	*Klebsiella pneumoniae*	NG_066711.1
26	GDA6P073	*Escherichia coli*	NG_067144.1
27	ST101	*Escherichia coli*	NG_062358.1
28	ASKPNKP22	*Klebsiella pneumoniae*	NG_064729.1
29	-	*Klebsiella pneumoniae*	NG_067145.1
30	KDCA201731	*Klebsiella oxytoca*	NG_071206.1
31	KDCA201761	*Citrobacter werkmanii*	NG_071207.1
34	Vb1473	*Vibrio parahaemolyticus*	NG_076661.1
35	N1949	*Escherichia coli*	NG_076662.1
36	JNQH462	*Escherichia coli*	NG_076641.1
37	JNQH497	*Escherichia coli*	NG_076642.1
38	2251506	*Providencia rettgeri*	NG_076664.1
39	01B18CPO004	*Klebsiella pneumoniae*	NG_076842.1
40	07C18CPO001	*Acinetobacter baumannii*	NG_076843.1
41	140507	*Klebsiella pneumoniae*	NG_078034.1

blaIMP	Strain Name	Organism Name	GenBank No.
1	PHB 2	*Pseudomonas aeruginosa*	GU831546.1
2	NCB405	*Serratia marcescens*	AB182996.1
3	JS19622	*Shigella flexneri*	NG_049194.1
4	-	*Acinetobacter baumannii*	NG_049203.1
5	65FFC	*Acinetobacter baumannii*	JF810083.1
6	E129	*Klebsiella pneumoniae*	AP019403.1
7	IMP699	*Pseudomonas aeruginosa*	AF416736.2
8	6FJ014	*Klebsiella pneumoniae*	EU368856.1
9	PA96	*Pseudomonas aeruginosa*	NG_049223.1
10	NPA1	*Pseudomonas aeruginosa*	AB195637.1
11	ABI1	*Acinetobacter baumannii*	NG_049174.1
12	758/00	*Pseudomonas putida*	NG_049175.1
13	86-14571A	*Pseudomonas aeruginosa*	NG_049176.1
14	-	*Pseudomonas aeruginosa*	NG_049177.1
15	-	*Pseudomonas aeruginosa*	NG_049178.1
16	101-4704C	*Pseudomonas aeruginosa*	NG_049179.1
17	Pa-7/02	*Pseudomonas aeruginosa*	NG_049180.1
18	-	*Pseudomonas aeruginosa*	NG_049181.1
19	PUJ-1	*Pseudomonas aeruginosa*	KF468750.1
20	MRY04-1316	*Pseudomonas aeruginosa*	NG_049184.1
21	NCB03-104	*Pseudomonas aeruginosa*	NG_049185.1
22	JICC 55398	*Providencia rettgeri*	AB754495.1
23	HSHCF-50	*Citrobacter freundii*	NG_049187.1
24	sm177-6	*Serratia marcescens*	NG_049188.1
25	KMP0701	*Pseudomonas aeruginosa*	NG_049189.1
26	DR26420/08	*Pseudomonas aeruginosa*	NG_049190.1
27	PmCB1	*Proteus mirabilis*	NG_049191.1
28	11005	*Klebsiella oxytoca*	NG_049192.1
29	BES10298	*Pseudomonas aeruginosa*	NG_049193.1
30	3107	*Pseudomonas aeruginosa*	NG_049195.1
31	NRZ-00156	*Pseudomonas aeruginosa*	NG_049196.1
32	KP-PNK-1	*Klebsiella pneumoniae*	NG_049197.1
33	85-7090A	*Pseudomonas aeruginosa*	NG_049198.1
34	MS5279	*Klebsiella oxytoca*	NG_049199.1
35	1876	*Pseudomonas aeruginosa*	NG_049200.1
37	CNR 06-BRE	*Pseudomonas aeruginosa*	NG_049201.1
38	A324	*Klebsiella pneumoniae*	MF344566.1
39	17-5136	*Pseudomonas aeruginosa*	NG_064724.1
40	JICC 55824	*Pseudomonas aeruginosa*	NG_049204.1
41	JICC 55826	*Pseudomonas aeruginosa*	NG_049205.1
42	JICC 55015	*Acinetobacter soli*	NG_049206.1
43	NCGM 1496	*Pseudomonas aeruginosa*	NG_049207.1
44	NCGM 1663	*Pseudomonas aeruginosa*	NG_049208.1
45	M140A	*Pseudomonas aeruginosa*	NG_049209.1
46	13-1694	*Pseudomonas putida*	NG_064725.1
47	894356	*Serratia marcescens*	KP050486.1
48	926467	*Pseudomonas aeruginosa*	NG_049210.1
49	1128657	*Pseudomonas aeruginosa*	NG_049211.1
51	NCGM 3025	*Pseudomonas aeruginosa*	NG_049213.1
52	TUM14831	*Escherichia coli*	NG_049214.1
53	NF812166	*Pseudomonas aeruginosa*	NG_049215.1
54	750109	*Pseudomonas aeruginosa*	NG_049216.1
55	56	*Acinetobacter baumannii*	NG_049217.1
56	1207312	*Pseudomonas aeruginosa*	NG_049218.1
58	CPO20150081	*Pseudomonas putida*	NG_049219.1
59	1216811	*Escherichia coli*	NG_055477.1
60	NCGM-ECI242	*Enterobacter cloacae*	NG_050945.1
61	NRZ-26903	*Acinetobacter baumannii*	NG_051166.1
62	PE83	*Pseudomonas aeruginosa*	NG_051513.1
63	1156	*Pseudomonas aeruginosa*	NG_052049.1
64	20299C	*Proteus mirabilis*	NG_054710.1
65	MPPA-138	*Pseudomonas aeruginosa*	NG_066508.1
66	NR336	*Escherichia coli*	NG_054676.1
67	Put_Imp_1	*Providencia rettgeri*	NG_055271.1
68	TA6363	*Klebsiella pneumoniae*	NG_055584.1
69	WCHPr-ZX09369	*Providencia sp.*	NG_055665.1
70	NR1418	*Providencia rettgeri*	LC348383.1
71	174324	*Pseudomonas aeruginosa*	NG_056414.1
72	1636483	*Pseudomonas aeruginosa*	MH021847.1
73	1739129	*Pseudomonas aeruginosa*	NG_057463.1
74	1714568	*Pseudomonas aeruginosa*	NG_057606.1
75	1641818	*Pseudomonas aeruginosa*	NG_057607.1
76	NCGM 3689	*Pseudomonas aeruginosa*	NG_061409.1
77	NCGM 3799	*Pseudomonas aeruginosa*	NG_061410.1
78	NCGM 3848	*Pseudomonas aeruginosa*	NG_061411.1
79	163874	*Pseudomonas aeruginosa*	NG_061626.1
80	NCGM 3336	*Pseudomonas aeruginosa*	NG_062274.1
81	1925992	*Pseudomonas aeruginosa*	MN267699.1
82	NRZ-43907	*Pseudomonas aeruginosa*	MN057782.1
83	1767579	*Pseudomonas aeruginosa*	MN104595.1
84	1849458	*Pseudomonas aeruginosa*	MN219692.1
85	197018	*Pseudomonas aeruginosa*	MN510335.1
86	KM2018-19	*Pseudomonas aeruginosa*	MT241520.1
87	KM2018-38	*Pseudomonas aeruginosa*	MT241521.1
88	JUPA4263	*Pseudomonas aeruginosa*	LC558310.1
89	420352	*Pseudomonas putida*	NG_070738.1
90	NRZ-62848	*Pseudomonas aeruginosa*	MW811441.1

blaVIM	Strain Name	Organism Name	GenBank No.
1	EYG91	*Proteus mirabilis*	JQ690541.1
2	DK45	*Pseudomonas aeruginosa*	GQ853417.1
3	PS-26/00	*Pseudomonas aeruginosa*	AF300454.1
4	-	*Pseudomonas aeruginosa*	AY702100.1
5	PSE K5	*Pseudomonas aeruginosa*	AY456196.1
6	DU25165/00	*Pseudomonas putida*	NG_050380.1
7	Pa2815	*Pseudomonas aeruginosa*	MF116160.1
8	Colombia PFGE type A	*Pseudomonas aeruginosa*	AY524987.1
9	UK 042	*Pseudomonas aeruginosa*	AY524988.1
10	UK 010	*Pseudomonas aeruginosa*	AY524989.1
11	M5109	*Pseudomonas aeruginosa*	AY605049.2
12	-	*Klebsiella pneumoniae*	DQ143913.1
13	-	*Pseudomonas aeruginosa*	NG_050340.1
14	PA210	*Pseudomonas aeruginosa*	FJ445404.1
15	9551	*Pseudomonas aeruginosa*	EU419745.1
16	166301	*Pseudomonas aeruginosa*	EU419746.1
17	D2816	*Pseudomonas aeruginosa*	EU118148.2
18	243-31-C	*Pseudomonas aeruginosa*	NG_050345.1
19	DIH-1	*Escherichia coli*	FJ822963.1
20	PA-VAL-A4	*Pseudomonas aeruginosa*	NG_050348.1
23	115-6196A	*Enterobacter cloacae*	GQ242167.1
24	Pa16	*Pseudomonas aeruginosa*	LC420030.1
25	P13	*Proteus mirabilis*	HM750249.1
26	Kpn1192	*Klebsiella pneumoniae*	KM975295.1
27	KPn254B	*Klebsiella pneumoniae*	HQ858608.1
28	PA 435	*Pseudomonas aeruginosa*	JF900599.1
29	957	*Escherichia coli*	NG_050355.1
30	11-527	*Pseudomonas aeruginosa*	NG_050357.1
31	11236	*Enterobacter cloacae*	JN982330.1
32	C4618	*Klebsiella oxytoca*	NG_050359.1
33	Kp7.11	*Klebsiella pneumoniae*	JX258134.1
34	K43	*Klebsiella pneumoniae*	JX185132.1
35	81-36520C	*Klebsiella oxytoca*	NG_050362.1
36	131-40581C	*Pseudomonas aeruginosa*	NG_050363.1
37	81-43486D	*Pseudomonas aeruginosa*	NG_050364.1
38	BP2	*Pseudomonas aeruginosa*	KC469971.2
39	Kpn7994	*Klebsiella pneumoniae*	KM975296.1
40	NMI8770/11	*Enterobacter cloacae*	MF678585.1
41	11310	*Pseudomonas aeruginosa*	KP771862.1
42	-	*Klebsiella pneumoniae*	NG_050370.1
43	-	*Pseudomonas aeruginosa*	NG_050371.1
44	1080816	*Pseudomonas aeruginosa*	NG_050372.1
45	1082670	*Pseudomonas aeruginosa*	NG_050373
46	15307	*Pseudomonas aeruginosa*	KP749829.1
47	PA-HSLI	*Pseudomonas aeruginosa*	KT954134.1
48	Tue2-1	*Citrobacter cronae*	KT964061.1
49	1266898	*Pseudomonas aeruginosa*	KU663374.1
50	1267499	*Pseudomonas aeruginosa*	KU663375.1
51	NRZ-24140	*Klebsiella pneumoniae*	KU746270.1
52	0593	*Klebsiella pneumoniae*	KX349731.1
53	VA-613-15	*Pseudomonas aeruginosa*	KX788872.1
54	NRZ-30617	*Serratia marcescens*	KY508061.1
55	BC-15-13	*Klebsiella pneumoniae*	MG552720.1
56	NRZ-38884	*Citrobacter freundii*	MG834535.1
57	PaTAN65	*Pseudomonas aeruginosa*	LC383983.1
58	NRZ-40481	*Enterobacter cloacae*	MH479908.1
59	207	*Citrobacter freundii*	MH548439.1
60	NCGM3750	*Pseudomonas aeruginosa*	NG_061404.1
61	ST27	*Pseudomonas aeruginosa*	MK098862.1
62	174364	*Pseudomonas putida*	MG717459.1
63	-	*Pseudomonas aeruginosa*	MK780742.1
64	NRZ-41827	*Klebsiella pneumoniae*	MK807022.1
65	NRZ-42039	*Citrobacter freundii*	MK807023.1
66	JUPA4001	*Pseudomonas aeruginosa*	NG_064786.1
67	INSAEclo26103	*Enterobacter hormaechei*	NG_065942.1
68	1776980	*Providencia stuartii*	MN267702.1
69	PAE1880	*Pseudomonas aeruginosa*	NG_067166.1
70	197069	*Pseudomonas aeruginosa*	MN920417.1
71	Vb1833	*Vibrio alginolyticus*	MT588301.1
72	1962327	*Pseudomonas aeruginosa*	MT872420.1
73	2122087	*Pseudomonas aeruginosa*	MT872421.1
74	NRZ-63987	*Pseudomonas aeruginosa*	MW811442.1
75	07C16CRGN002	*Proteus mirabilis*	MZ748327.1
76	JUPA4312	*Pseudomonas aeruginosa*	LC648428.1
77	4147/15	*Pseudomonas shirazica*	MZ947163.1
78	NRZ-68977	*Proteus mirabilis*	OK180983.1
79	NMI1536_13	*Klebsiella oxytoca*	OK217280.1

Table S2. Primers used to sequence the LAMP products.

Primer name	Primer Sequence (Sequence 5'-3')
KPC_F2	TGT AAG TTA CCG CGC TGA GG
KPC_B2	TTT TCC GAG ATG GGT GAC CAC
NDM-1_F2	CGA CGA TTG GCC AGC AAA T
NDM-1_B2	GCC ATC CCT GAC GAT CAA AC
IMP-1_F2	CGT AGT GGT TTG GTT GCC TG
IMP-1_B2	TGG AAC AAC CAG TTT TGC CTT A
VIM-2_F2	GGG TGC GAA AAA CAC AGC
VIM-2_B2	GCT GAT GCG TAC GTT GCC

Table S3. PCR primers for the β-lactamase genes.

Target	Primer name	Conventional PCR primer (Sequence 5'-3')	Amplicon size (bp)	Reference
bla$_{KPC}$	KPC-F	TCG CTA AAC TCG AAC AGG	785	Monteiro et al., 2012
	KPC-R	TTA CTG CCC GTT GAC GCC CAA TCC		
bla$_{NDM-1}$	NDM-F	TTG GCC TTG CTG TCC TTG	82	
	NDM-R	ACA CCA GTG ACA ATA TCA CCG		
bla$_{VIM}$	VIM-F	GTT TGG TCG CAT ATC GCA AC	382	
	VIM-R	AAT GCG CAG CAC CAG GAT AG		
bla$_{IMP}$	IMP-F	CTA CCG CAG CAG AGT CTT TG	587	Senda et al., 1996
	IMP-R	AAC CAG TTT TGC CTT ACC AT		
