# Supplementary material for: Development of a novel loop-mediated isothermal amplification assay for ß-lactamase gene identification using clinical isolates of Gram-negative bacteria
Source: Front Cell Infect Microbiol. 2023 Jan 12;12:1000445. doi: 10.3389/fcimb.2022.1000445 (PMC9877619; doi:10.3389/fcimb.2022.1000445)
Supplement: Supplementary file 2 [file DataSheet_2.pdf]

Figure S1. Multiple sequence alignment of variants of (a) *bla*$_{KPC}$, (b) *bla*$_{NDM}$, (c) *bla*$_{IMP}$, and (d) *bla*$_{VIM}$, using Clustal X v. 2. Asterisk, consensus sequence; bar chart, rate of consensus sequences against each alignment column.

(a) *bla*$_{KPC}$

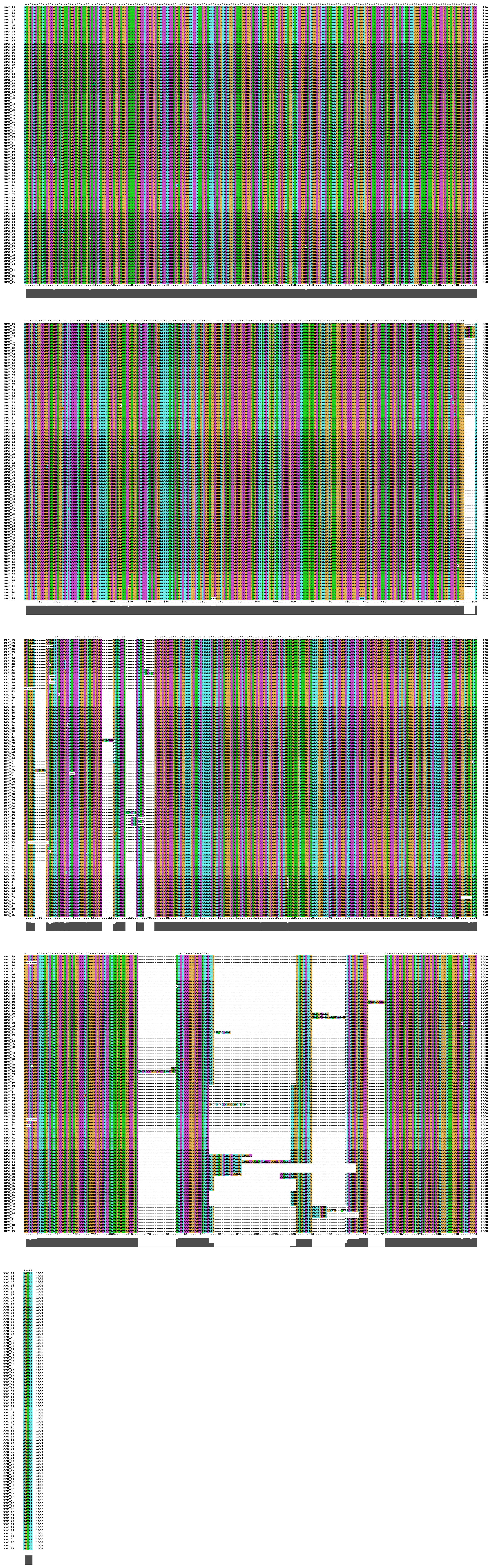

(b) bla_{NDM}

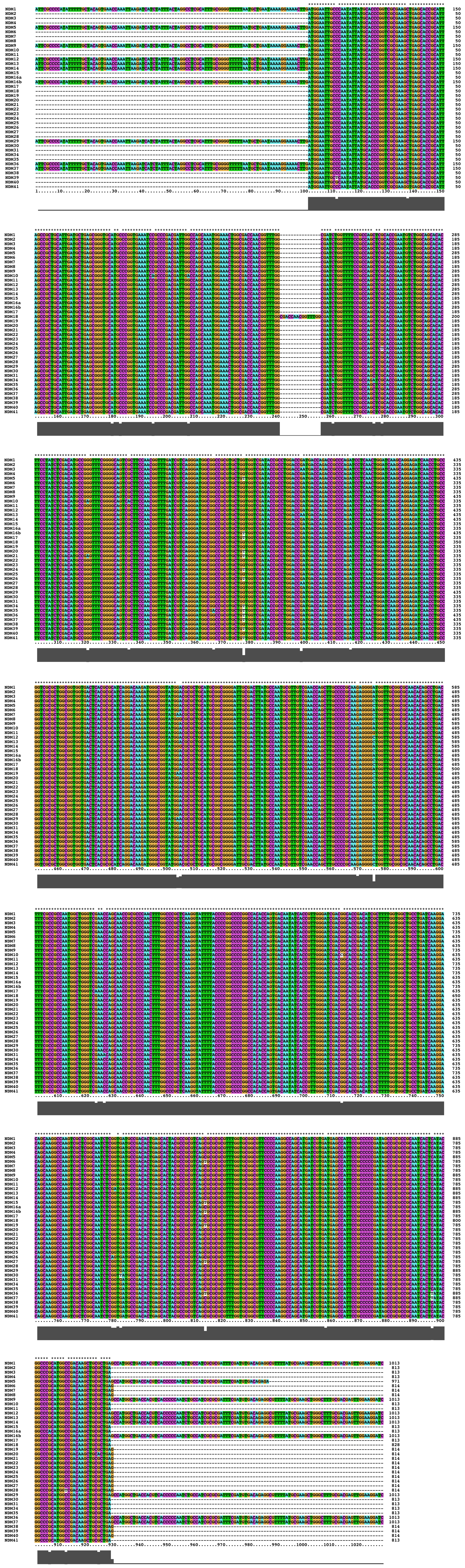

(c) bla_{IMP}

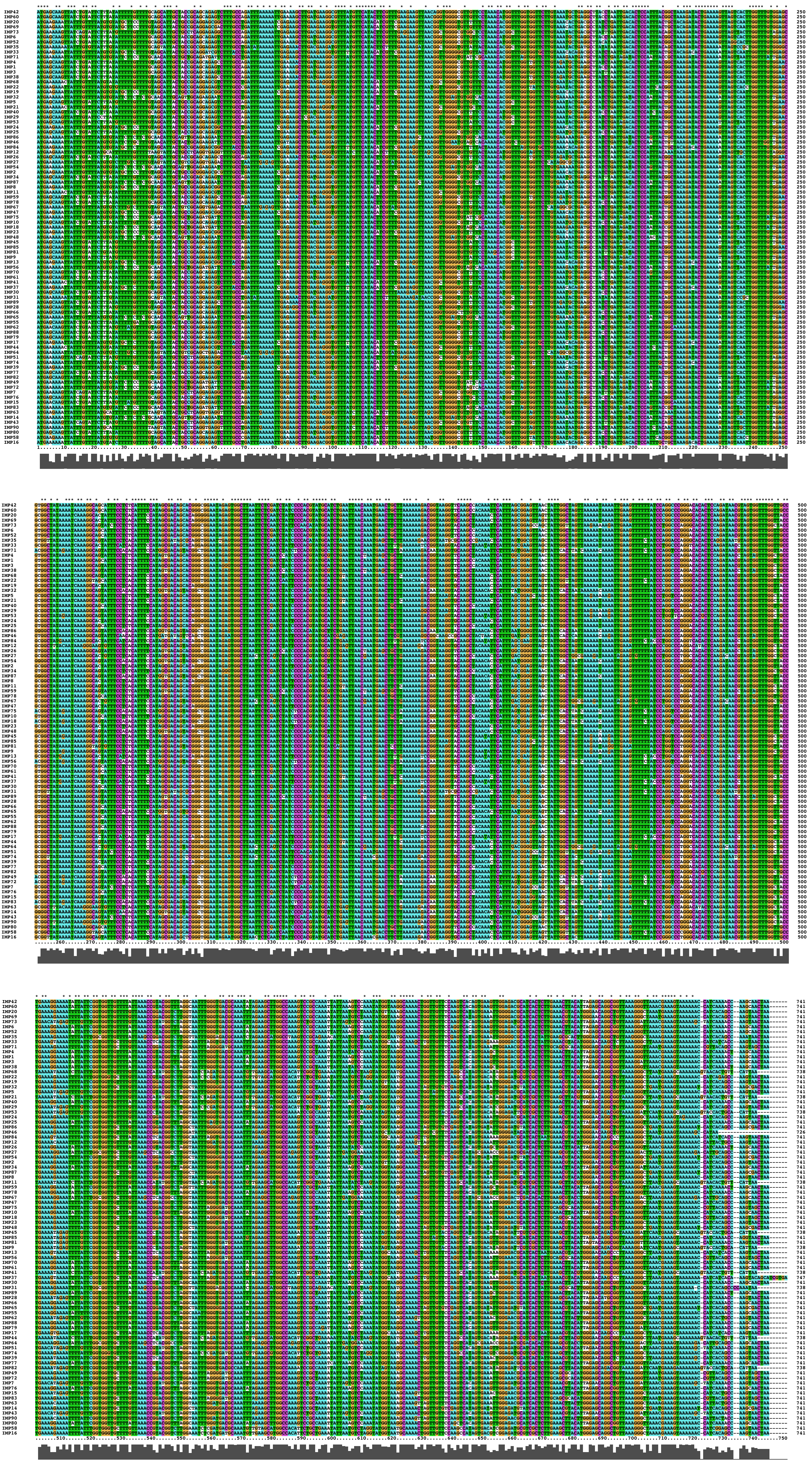

(d) *bla*$_{VIM}$

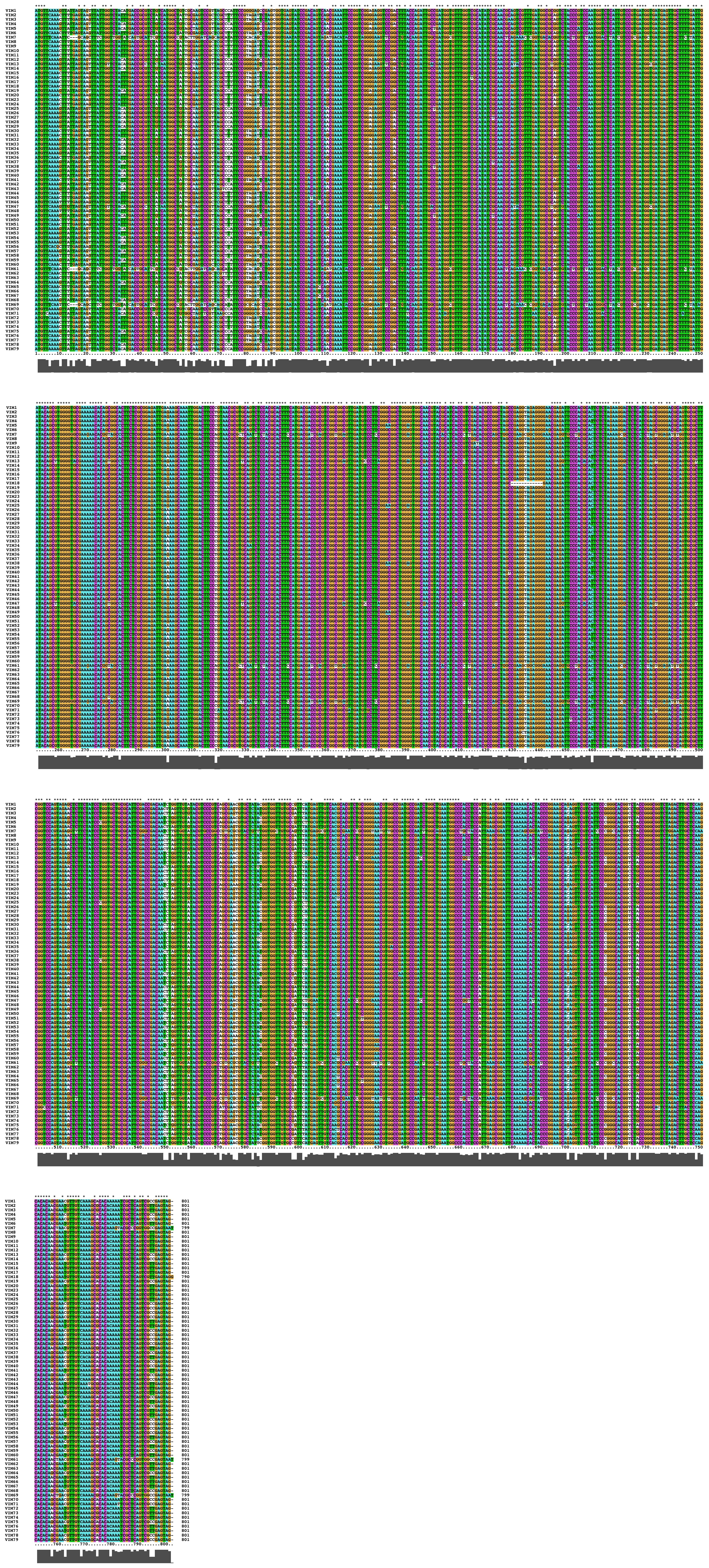

Figure S2. Nucleotide sequences of the four β-lactamase genes (*bla*$_{KPC}$, *bla*$_{NDM-1}$, *bla*$_{IMP-1}$ group, and *bla*$_{VIM}$) used to design the LAMP primer sets. Arrows, LAMP primer sequences. (a–d) *bla*$_{KPC}$, *bla*$_{NDM-1}$, *bla*$_{IMP-1}$ group, and *bla*$_{VIM}$ sequences, respectively.

(a) *bla*$_{KPC-2}$, GeneBank no. NG_049253.1

```
No.         1                                                                         70
sequence    CGTTGATGTC ACTGTATCGC CGTCTAGTTC TGCTGTCTTG TCTCTCATGG CCGCTGGCTG GCTTTTCTGC


No.         71                                                                       140
sequence    CACCGCGCTG ACCAACCTCG TCGCGGAACC ATTCGCTAAA CTCGAACAGG ACTTTGGCGG CTCCATCGGT

No.         141                                                                      210
sequence    GTGTACGCGA TGGATACCGG CTCAGGCGCA ACTGTAAGTT ACCGCGCTGA GGAGCGCTTC CCACTGTGCA
primer      |--------F3--------->              |--------F2---------->               <--

No.         211                                                                      280
sequence    GCTCATTCAA GGGCTTTCTT GCTGCCGCTG TGCTGGCTCG CAGCCAGCAG CAGGCCGGCT TGCTGGACAC
primer      ----LF--------|<--------F1-----------|                       |----B1--------

No.         281                                                                      350
sequence    ACCCATCCGT TACGGCAAAA ATGCGCTGGT TCCGTGGTCA CCCATCTCGG AAAAATATCT GACAACAGGC
primer      -----> |--------LB-------->         <--------B2----------|            <-----

No.         351                                                                      420
Sequence    ATGACGGTGG CGGAGCTGTC CGCGGCCGCC GTGCAATACA GTGATAACGC CGCCGCCAAT TTGTTGCTGA
primer      B3 -----|

No.         421                                                                      490
Sequence    AGGAGTTGGG CGGCCCGGCC GGGCTGACGG CCTTCATGCG CTCTATCGGC GATACCACGT TCCGTCTGGA


No.         491                                                                      560
Sequence    CCGCTGGGAG CTGGAGCTGA ACTCCGCCAT CCCAGGCGAT GCGCGCGATA CCTCATCGCC GCGCGCCGTG


No.         561                                                                      630
Sequence    ACGGAAAGCT TACAAAAACT GACACTGGGC TCTGCACTGG CTGCGCCGCA GCGGCAGCAG TTTGTTGATT
```

(b) *bla*$_{NDM-1}$, GeneBank no. FN396876.1

```
No.        1                                                                     70
sequence   ATGGAATTGC CCAATATTAT GCACCCGGTC GCGAAGCTGA GCACCGCATT AGCCGCTGCA TTGATGCTGA


No.        71                                                                   140
Sequence   GCGGGTGCAT GCCCGGTGAA ATCCGCCCGA CGATTGGCCA GCAAATGGAA ACTGGCGACC AACGGTTTGG
primer          |--------F3--------->   |--------F2--------->  <--------LF---------|

No.        141                                                                  210
sequence   CGATCTGGTT TTCCGCCAGC TCGCACCGAA TGTCTGGCAG CACACTTCCT ATCTCGACAT GCCGGGTTTC
primer       <--------F1----------|       |--------B1---------->                |----

No.        211                                                                  280
sequence   GGGGCAGTCG CTTCCAACGG TTTGATCGTC AGGGATGGCG GCCGCGTGCT GGTGGTCGAT ACCGCCTGGA
primer     ---LB-------->      <--------B2----------|                    <------------

No.        281                                                                  350
sequence   CCGATGACCA GACCGCCCAG ATCCTCAACT GGATCAAGCA GGAGATCAAC CTGCCGGTCG CGCTGGCGGT
primer     B3----|

No.        351                                                                  420
Sequence   GGTGACTCAC GCGCATCAGG ACAAGATGGG CGGTATGGAC GCGCTGCATG CGGCGGGGAT TGCGACTTAT

No.        421                                                                  490
Sequence   GCCAATGCGT TGTCGAACCA GCTTGCCCCG CAAGAGGGGA TGGTTGCGGC GCAACACAGC CTGACTTTCG

No.        491                                                                  560
Sequence   CCGCCAATGG CTGGGTCGAA CCAGCAACCG CGCCCAACTT TGGCCCGCTC AAGGTATTTT ACCCCGGCCC

No.        561                                                                  630
Sequence   CGGCCACACC AGTGACAATA TCACCGTTGG GATCGACGGC ACCGACATCG CTTTTGGTGG CTGCCTGATC
```

(c) *bla*$_{IMP-1}$, GeneBank no. GU831546.1

```
No.       71                                                                       140
sequence  ATTTAAAAAT TGAAAAGCTT GATGAAGGCG TTTATGTTCA TACTTCGTTT GAAGAAGTTA ACGGGTGGGG

No.       141                                                                      210
sequence  CGTTGTTCCT AAACATGGTT TGGTGGTTCT TGTAAATGCT GAGGCTTACC TAATTGACAC TCCATTTACG

No.       211                                                                      280
sequence  GCTAAAGATA CTGAAAAGTT AGTCACTTGG TTTGTGGAGC GTGGCTATAA AATAAAAGGC AGCATTTCCT

No.       281                                                                      350
sequence  CTCATTTTCA TAGCGACAGC ACGGGCGGAA TAGAGTGGCT TAATTCTCGA TCTATCCCCA CGTATGCATC

No.       351                                                                      420
sequence  TGAATTAACA AATGAACTGC TTAAAAAAGA CGGTAAGGTT CAAGCCACAA ATTCATTTAG CGGAGTTAAC

No.       421                                                                      490
Sequence  TATTGGCTAG TTAAAAATAA AATTGAAGTT TTTTATCCAG GCCCGGGACA CACTCCAGAT AACGTAGTGG
primer                                                  |--------F3------->   |-------

No.       491                                                                      560
Sequence  TTTGGTTGCC TGAAAGGAAA ATATTATTCG GTGGTTGTTT TATTAAACCG TACGGTTTAG GCAATTTGGG
primer    F2---------->  <---------LF---------|                   <-------F1----------

No.       561                                                                      630
Sequence  TGACGCAAAT ATAGAAGCTT GGCCAAAGTC CGCCAAATTA TTAAAGTCCA AATATGGTAA GGCAAAACTG
primer    -|           |--------B1---------->|-----------LB------------><--------B2---

No.       631                                                                      700
Sequence  GTTGTTCCAA GTCACAGTGA AGTTGGAGAC GCATCACTCT TGAAACTTAC ATTAGAGCAG GCGGTTAAAG
primer    --------|                 <--------B3------------|
```

(d) ***bla*$_{\mathbf{VIM-2}}$, GeneBank no. GQ 853417.1**

```
No.         1                                                                         70
sequence    ATGTTCAAAC TTTTGAGTAA GTTATTGGTC TATTTGACCG CGTCTATCAT GGCTATTGCG AGTCCGCTCG


No.         71                                                                       140
Sequence    CTTTTTCCGT AGATTCTAGC GGTGAGTATC CGACAGTCAG CGAAATTCCG GTCGGGGAGG TCCGGCTTTA


No.         141                                                                      210
sequence    CCAGATTGCC GATGGTGTTT GGTCGCATAT CGCAACGCAG TCGTTTGATG GCGCAGTCTA CCCGTCCAAT

No.         211                                                                      280
sequence    GGTCTCATTG TCCGTGATGG TGATGAGTTG CTTTTGATTG ATACAGCGTG GGGTGCGAAA AACACAGCGG
primer                   |---------F3---------->                    |-------F2-------->

No.         281                                                                      350
sequence    CACTTCTCGC GGAGATTGAG AAGCAAATTG GACTTCCTGT AACGCGTGCA GTCTCCACGC ACTTTCATGA
primer               <--------LF--------|<--------F1---------|       |--------B1--------

No.         351                                                                      420
Sequence    CGACCGCGTC GGCGGCGTTG ATGTCCTTCG GGCGGCTGGG GTGGCAACGT ACGCATCAGC GTCGACACGC
primer      ->                |-------LB------->          <---------B2------|

No.         421                                                                      490
Sequence    CGGCTAGCCG AGGTAGAGGG GAACGAGATT CCCACGCACT CTCTAGAAGG ACTCTCATCG AGCGGGGACG
primer              <--------B3--------|

No.         491                                                                      560
Sequence    CAGTGCGCTT CGGTCCAGTA GAACTCTTCT ATCCTGGTGC TGCGCATTCG ACCGACAACT TAGTTGTGTA

No.         561                                                                      630
Sequence    CGTCCCGTCT GCGAGTGTGC TCTATGGTGG TTGTGCGATT TATGAGTTGT CACGCACGTC TGCGGGGAAC
```

Figure S3. Phylogenetic analysis using the primer region sequences of variants of the four β-lactamase genes. (a–d) bla_{KPC}, $bla_{\text{NDM-1}}$, $bla_{\text{IMP-1}}$ group, and bla_{VIM}, respectively. Alignments (a-1, b-1, c-1, and d-1) were conducted using Clustal W in MEGA v. 11 (Tamura et al., 2021). Capital letter, point mutation; dot (.), matched nucleotide. Phylogenetic trees (a-2, b-2, c-2, and d-2) were constructed by the neighbor-joining (NJ) method in MEGA v. 11 (Tamura et al., 2021). Blue square, anticipated detectable variants.

(a-1) bla_{KPC}

KPC gene types and sequence	F3	F2	LF	F1
KPC2[a]	TGTACGCGATGGATACCGG	TGTAAGTTACCGCGCTGAGG	AGCTCATTCAAGGGC	TTTCTTGCTGCCGCTGTGCTG
KPC54	....	.............T......	....	....
KPC21[b]	....	....	....	....
KPC22	....	....	....	....
KPC45	....	....	....	....
KPC15	....	....	....	....
KPC59	....	....	....	....
KPC7	..........A........	....	....	....
KPC13	....	....	....	....

KPC gene types and sequence	B1	LB	B2	B3
KPC2[a]	GGCTTGCTGGACACACCCAT	CGTTACGGCAAAAATGCGC	TGGTCACCCATCTCGGAAAA	ACAACAGGCATGACGGTG
KPC54	....	....	....	....
KPC21[b]	....	....	A...................	....
KPC22	....	....	G...................	....
KPC45	.............A......	....	....	....
KPC15	....	....	....	.................T
KPC59	.A..................	....	....	....
KPC7	....	....	....	....
KPC13	..........G.........	....	....	....

KPC2[a]; KPC-2, 3-6, 8, 10-12, 14, 16-20, 23-26, 28-44, 46-53, 55-58, 60-82, 84-88, 90, 91, and 94-98: KPC21[b]; KPC-21 and 27.

(a-2) *bla*$_{\mathbf{KPC}}$.

The LAMP primers for *bla*$_{KPC}$ detected *bla*$_{KPC-2}$ and *bla*$_{KPC-3}$.

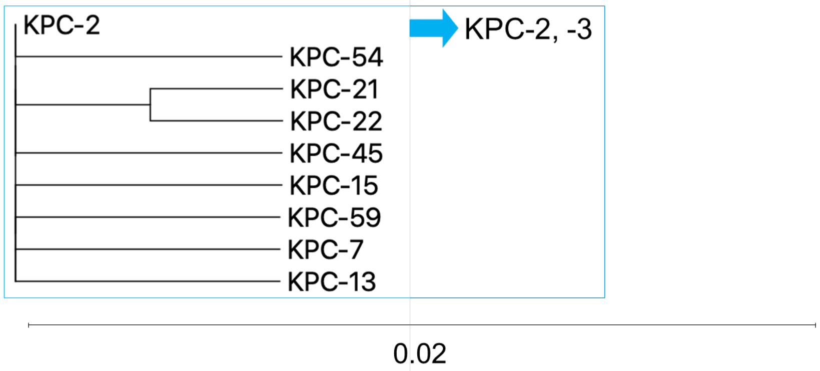

KPC-2; KPC-2, 3-6, 8, 10-12, 14, 16-20, 23-26, 28-44, 46-53, 55-58, 60-82, 84-88, 90, 91, and 94-98: KPC-21; KPC-21 and 27.

(b-1) *bla*$_{NDM}$

NDM gene types and sequence	F3	F2	LF	F1
NDM1[a]	TGCATGCCCGGTGAAATCC	CGACGATTGGCCAGCAAAT	AAACTGGCGACCAACGGTT	ATCTGGTTTTCCGCCAGCTC
NDM2	......G............	....	....	....
NDM3[b]	....	....	....	....
NDM38	...G...............	....	....	....
NDM34	....	....	....	..A..............A..
NDM10	..................A	.........A.........	....	....

NDM gene types and sequence	B1	LB	B2	B3
NDM1[a]	ATGTCTGGCAGCACACTTCC	TTTCGGGGCAGTCGCTT	GTTTGATCGTCAGGGATGGC	ATACCGCCTGGACCGATGA
NDM2	....	....	....	....
NDM3[b]	....	....	....	..............A....
NDM38	....	....	....	....
NDM34	....	....	....	....
NDM10	....	.............A...	....	....

NDM1[a]; NDM-1, 4-9, 11, 12, 14, 15, 16a, 16b, 17-26, 28-31, 35-37, and 39-41: NDM3[b]; NDM-3, 13, and 27.

(b-2) *bla*$_{\mathbf{NDM}}$

The LAMP primers for *bla*$_{NDM-1}$ detected *bla*$_{NDM-1}$.

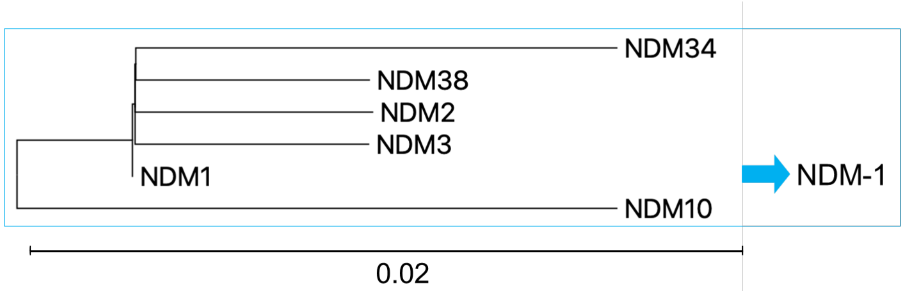

NDM1; NDM-1, 4-9, 11, 12, 14, 15, 16a, 16b, 17-26, 28-31, 35-37, and 39-41: NDM3; NDM-3, 13, and 27.

(c-1) *bla*$_{IMP}$

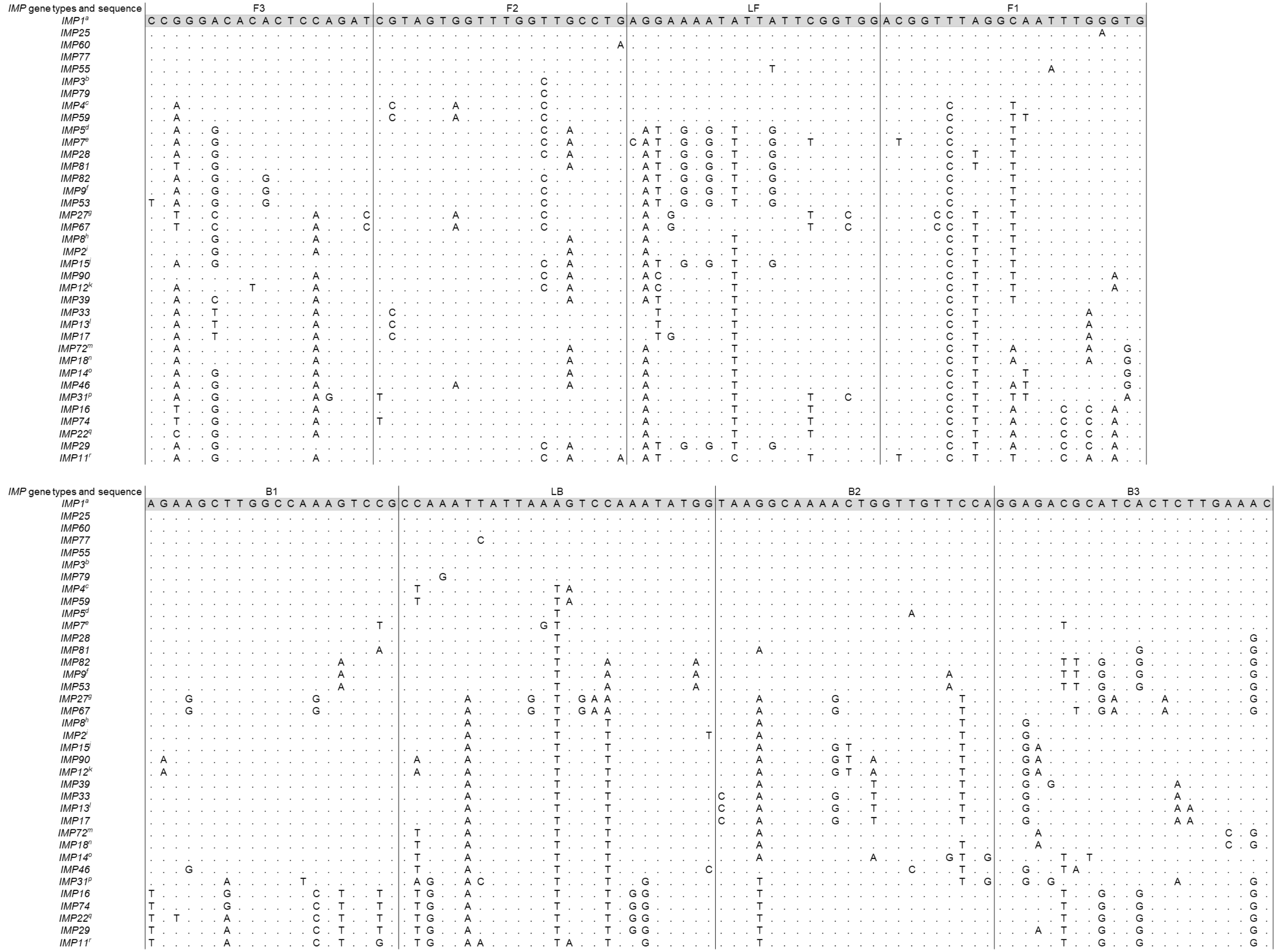

IMP1[a]; IMP-1, 6, 10, 30, 40, 42, 52, 61, 66, 70, 76, 78, 80, and 88: IMP3[b]; IMP-3 and 34: IMP4[c]; IMP-4, 26, 38, and 89: IMP5[d]; IMP-5 and 85: IMP7[e]; IMP-7, 43, 51, and 73: IMP9[f]; IMP-9 and 45: IMP27[g]; IMP-27 and 64: IMP8[h]; IMP-8, 23, 24, 47, and 69: IMP2[i]; IMP-2, 19, and 20: IMP15[j]; IMP-15 and 62: IMP12[k]; IMP-12 and 63: IMP13[l]; IMP-13, 37, and 84: IMP72[m]; IMP-72 and 75: IMP18[n]; IMP-18, 49, 56, 71, and 83: IMP14[o]; IMP-14, 32, 48, 54, 65, 86, and 87: IMP31[p]; IMP-31 and 35: IMP22[q]; IMP-22 and 58: IMP11[r]; IMP-11, 21, 41, 44, and 68.

(c-2) *bla*$_{IMP}$

Using the LAMP primers for the *bla*$_{IMP-1}$ group, *bla*$_{IMP-1}$ and *bla*$_{IMP-4}$ were detected but *bla*$_{IMP-13}$, *bla*$_{IMP-15}$, *bla*$_{IMP-18}$ were not.

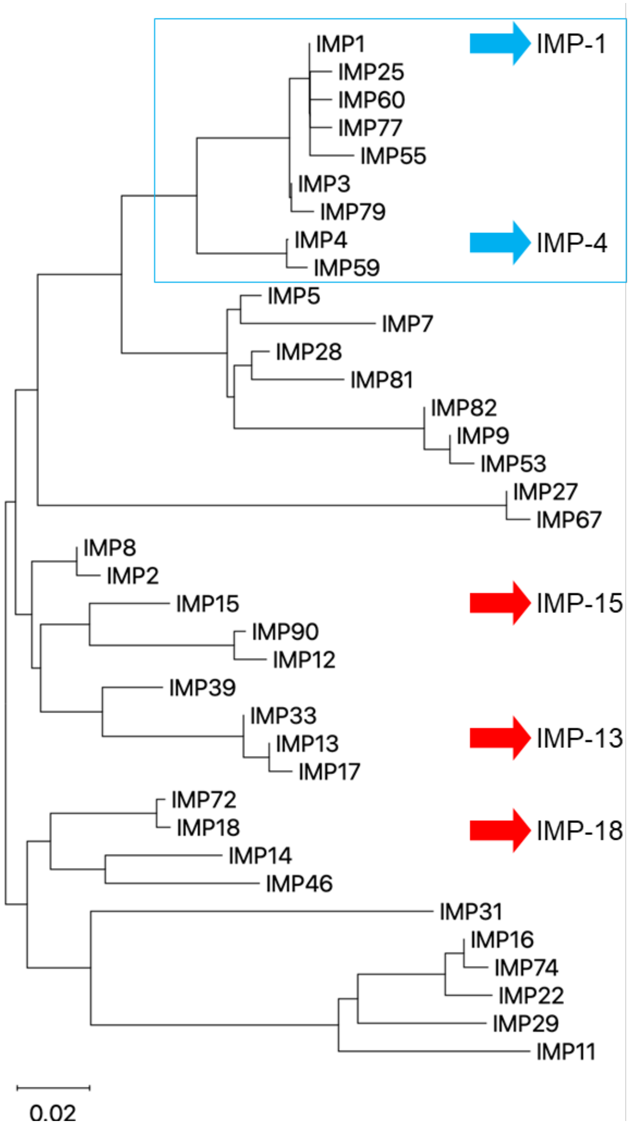

IMP1; IMP-1, 6, 10, 30, 40, 42, 52, 61, 66, 70, 76, 78, 80, and 88: IMP3; IMP-3 and 34: IMP4; IMP-4, 26, 38, and 89: IMP5; IMP-5 and 85: IMP7; IMP-7, 43, 51, and 73: IMP9; IMP-9 and 45: IMP27; IMP-27 and 64: IMP8; IMP-8, 23, 24, 47, and 69: IMP2; IMP-2, 19, and 20: IMP15; IMP-15 and 62: IMP12; IMP-12 and 63: IMP13; IMP-13, 37, and 84: IMP72; IMP-72 and 75: IMP18; IMP-18, 49, 56, 71, and 83: IMP14; IMP-14, 32, 48, 54, 65, 86, and 87: IMP31; IMP-31 and 35: IMP22; IMP-22 and 58: IMP11; IMP-11, 21, 41, 44, and 68.

(d-1) *bla*$_{VIM}$

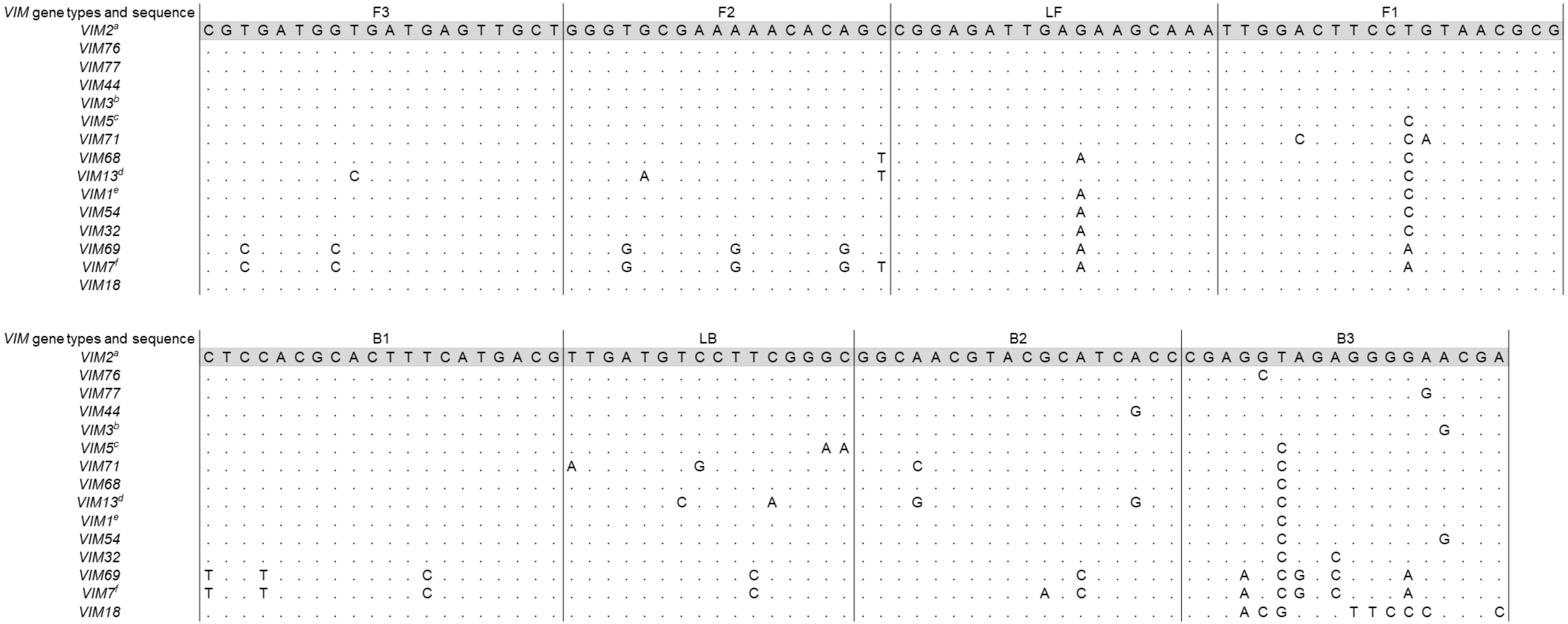

VIM2[a]; VIM-2, 8-10, 15-17, 20, 23, 24, 30, 31, 36, 41, 45, 46, 48, 51, 53, 56, 58, 60, 62, 63, 65-67, and 72-74: VIM3[b]; VIM-3, 6, 11, and 50: VIM13[c]; VIM-13 and 47: VIM5[d]; VIM-5, 25, 38, and 49: VIM1[e]; VIM-1, 4, 12, 14, 19. 26-29, 33-35, 37, 39, 40, 42, 43, 52, 55, 57, 59, 64, 70, 75, 78, and 79: VIM7[f]; VIM-7 and 61

(d-2) *bla*$_{VIM}$

The LAMP primers for *bla*$_{VIM}$ detected *bla*$_{VIM-1, 2, 4, 5, and 11}$, but not *bla*$_{VIM-7}$.

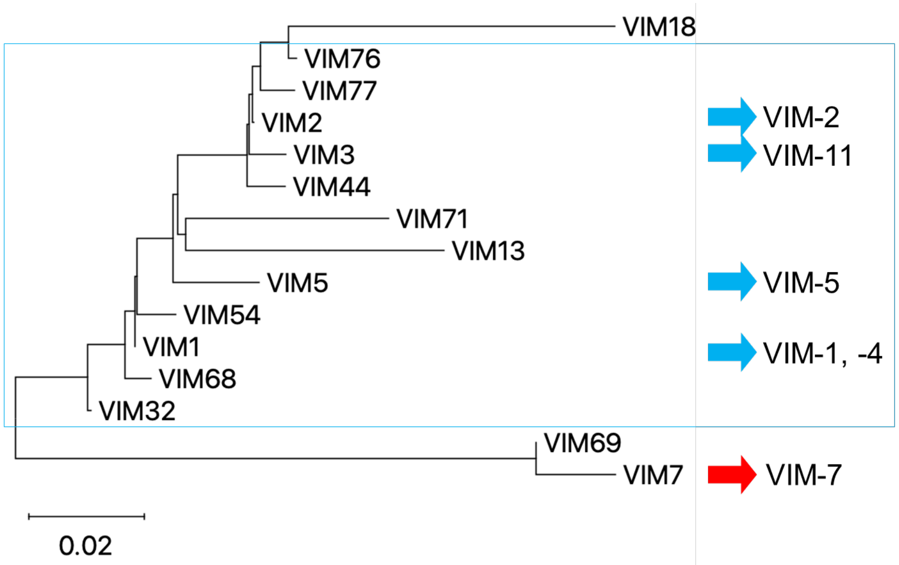

VIM2; VIM-2, 8-10, 15-17, 20, 23, 24, 30, 31, 36, 41, 45, 46, 48, 51, 53, 56, 58, 60, 62, 63, 65-67, and 72-74: VIM3; VIM-3, 6, 11, and 50: VIM13[c]; VIM-13 and 47: VIM5; VIM-5, 25, 38, and 49: VIM1; VIM-1, 4, 12, 14, 19. 26-29, 33-35, 37, 39, 40, 42, 43, 52, 55, 57, 59, 64, 70, 75, 78, and 79: VIM7; VIM-7 and 61

Figure S4. Sequences of the LAMP products for the ß-lactamase genes.

Sequences of the products of (a) $\mathit{bla}_{\mathrm{KPC}}$, (b) $\mathit{bla}_{\mathrm{NDM\text{-}1}}$, (c) $\mathit{bla}_{\mathrm{IMP\text{-}1}}$ group, and (d) $\mathit{bla}_{\mathrm{VIM}}$.

(a)

TTTCTTGCTG CCGCTGTGCT GGCTCGCAGC CAGCAGCAGG CCGGCTTGCT GGACACACCC AT

(b)

ATCTGGTTTT CCGCCAGCTC GCACCGAATG TCTGGCAGCA CACTTCC

(c)

ACGGTTTAGG CAATTTGGGT GACGCAAATA TAGAAGCTTG GCCAAAGTCC G

(d)

TTGGACTTCC TGTAACGCGT GCAGTCTCCA CGCACTTTCA TGACG
